# Tracing geochemical sources and health risk assessment of uranium in groundwater of arid zone of India

**DOI:** 10.1038/s41598-022-05770-2

**Published:** 2022-06-01

**Authors:** P. Pandit, Atul Saini, Sabarathinam Chidambaram, Vinod Kumar, Banjarani Panda, A. L. Ramanathan, Netrananda Sahu, A. K. Singh, Rohit Mehra

**Affiliations:** 1 USICT, GGSIPU, Dwarka, Delhi, 110078 India; 2 Department of Geography, Delhi School of Economics, University of Delhi, Delhi, 110007 India; 3 Present Address: Delhi School of Climate Change & Sustainability, Institution of Eminence, University of Delhi, Delhi, 110007 India; 4 Water Research Center, Kuwait Institute for Scientific Research, Kuwait City, Kuwait; 5 Department of Botany, Government Degree College, Ramban, Jammu, 182144 India; 6 Water Sciences Lab, University of Nebraska-Lincoln, Lincoln, NE USA; 7 School of Environmental Sciences, Jawahar Lal Nehru University, New Delhi, 110067 India; 8 Maharaja Surajmal Institute of Technology, USICT, GGSIPU, New Delhi, 110058 India; 9 B. R. Ambedkar National Institute of Technology, Jalandhar, 144011 India

**Keywords:** Environmental chemistry, Environmental impact

## Abstract

Water quality degradation and metal contamination in groundwater are serious concerns in an arid region with scanty water resources. This study aimed at evaluating the source of uranium (U) and potential health risk assessment in groundwater of the arid region of western Rajasthan and northern Gujarat. The probable source of vanadium (V) and fluorine (F) was also identified. U and trace metal concentration, along with physicochemical characteristics were determined for 265 groundwater samples collected from groundwater of duricrusts and palaeochannels of western Rajasthan and northern Gujarat. The U concentration ranged between 0.6 and 260 μg L^−1^ with a mean value of 24 μg L^−1^, and 30% of samples surpassed the World Health Organization’s limit for U (30 μg L^−1^). Speciation results suggested that dissolution of primary U mineral, carnotite [K_2_(UO_2_)_2_(VO_4_)_2_·3H_2_O] governs the enrichment. Water–rock interaction and evaporation are found the major hydrogeochemical processes controlling U mineralization. Groundwater zones having high U concentrations are characterized by Na–Cl hydrogeochemical facies and high total dissolved solids. It is inferred from geochemical modelling and principal component analysis that silicate weathering, bicarbonate complexation, carnotite dissolution, and ion exchange are principal factors controlling major solute ion chemistry. The annual ingestion doses of U for all the age groups are found to be safe and below the permissible limit in all samples. The health risk assessment with trace elements manifested high carcinogenic risks for children.

## Introduction

Groundwater accounts for 70% of the domestic, irrigation, and industrial use^1^ in an arid ecosystem, such as Rajasthan^2^. Calcrete hosted carnotite ([﻿K_2_(UO_2_)_2_(VO_4_)_2_·3H_2_O]; hydrated potassium uranium (U) vanadate) mineralization occurs in districts of western Rajasthan and northern Gujarat in fluvial to lacustrine and/or playa system, which has not been subjected yet to significant burial.

Calcretes (Silcrete, Ferricrete, Gypcrete) are normally terrestrial near-surface secondary calcium carbonate (CaCO_3_) accumulation in the soil profile, bedrock, and sediments^3^. These calcrete/ gypcrete sediments are the host for surficial-type U deposits which are the youngest deposits among late Tertiary-Quaternary ages^4^. Numerous biochemical and hydrogeochemical processes, such as evaporation, mineralization, intermixing with salty water, chloritization and sericitization lead to a change in the composition of the groundwater. Globally calcretes constitute 13% of the continental area and form an important part of the ecosystem. This leads to the concentration of multiple elements in the groundwater. The co-occurrence of multiple contaminants, coupled with overexploitation and the regional decline has led to severe water scarcity. Water quality degradation and U contamination in groundwater in India are known major concerns in the current scenario^5^. Numerous studies have been focused on groundwater depletion and contamination in India^6–8^. Episodes of high U, nitrate (NO_3_^2−^), F^−^ and arsenic (As) contamination are reported in several studies^9–11^. While WHO (1999) has set a provisional guideline of 30 μg L^−1^ for U concentration in drinking water, and the Atomic Energy Regulatory Board (AERB)^12^ in 2004 has set up a radiological limit of 60 μg L^−1^, no such limit has yet been set by the Bureau of Indian Standards (BIS)^13^. The upper limit of concentration of heavy metals and physicochemical parameters in groundwater has also been limited by the WHO^14^ and the BIS^13^. It is worthy to mention that the hydrogeochemical approach is essential to delineate the origin of U mineralization. Besides this, the role of hydrogeochemistry and geology have already been explored in the arid regions related to the evapotranspiration process in the tropical areas of Langer Heinrich (Southern Hemisphere), Klein Trekkopje (Namibia), Yeelirrie (Western Australia), Lake Maitland (southern Argentina) and Latin America^15–19^. The seasonal variations and the process of Uranium mobilisations in hard rock aquifers along with their health hazard indices in the south Indian aquifer indicated the redox reaction as a major governing factor^20,21^. However hydrogeochemical and U databases in groundwater of the palaeochannels in the chemical delta distributed in parts of Thar Desert of the western Rajasthan and northern Gujarat are meagre. Therefore, an attempt is made to create a geochemical database of the heavy metals and physicochemical parameters in the groundwater of the arid region of Rajasthan and Gujarat. The study becomes significant in itself because the groundwater serves as the main source for domestic and irrigation purposes in this region.

The objectives of this study are as follows: (1) to identify the major hydogeochemical process associated with U mineralization in calcrete groundwaters along the palaeochannels of the Luni river system; (2) to identify the main source of U; and (3) to assess the spatial distribution and health risk assessment of U. Results of this study would be useful in framing policies to regulate water quality.

## Material and methods

### Study area and geology

The study area is bounded by latitudes 24°–29° N and longitudes 70°–76° E, covering the western part of Rajasthan and northern part of Gujarat, India. The sampling areas included the palaeodrainages of Luni, Khari bani, Jojori river, Sukri, Mithri, Sagi, and Guhiya rivers in Nagaur, Pali, Jalore, Sirohi, and Banaskantha districts of Rajasthan and Gujarat. Samples were collected from palaeochannels and groundwater from wells, tube-wells and taps. The sampling area falls in the arid and semi-arid region of the Thar desert along the carnotite mineralization area. The average rainfall in Pali district is 462 mm; whereas in Nagaur district, it is 310 mm. Barmer lies in the arid region with an average annual rainfall of 260 mm and potential evaporation of 1857 mm. In Jalore and Sirohi districts, the average rainfall is 280 mm and 606 mm. Luni is the major drainage system in western Rajasthan with many tributaries like Sukri, Sagi, Bandi, and Jawai. Guhiya river originates near Khariyaniv and Tharasani villages in Pali District in the hillocks. These palaeochannels drain through Malani Igneous Suite (MIS) especially Jalore granites and are located in the northwestern part of Rajasthan. These granites are adjacent to the paleochannels of late Tertiary to Quaternary settings; while a small portion belongs to Neoproterozoic, Paleoproterozoic, and Mesoproterozoic age^22^. The water table depths ranged from 50 to 150 m in the current study area^23^. In India, a favourable geological and climatic setup is evident in western Rajasthan and Northern Gujarat for the formation of surficial-type of U mineralization. The area under investigation is mostly covered with Quaternary formations concealing Sirohi, Erinpura, and MIS rocks. Calcrete occurrences along western Rajasthan and Northern Gujarat are well-known due to their arid climatic conditions, and these are indicators of palaeo-climatic conditions. The vast development of calcretes spreads over 0.32 million m^2^. Fluorite (CaF_2_) and Barite (BaSO_4_) occur in the form of veins in volcanic agglomerates. The Department of Mines and Geology, Government of Rajasthan has estimated fluorite reserves of 0.17 million tonnes in the Jalore area.

### Groundwater sampling and chemical analysis

**Figure 1:**
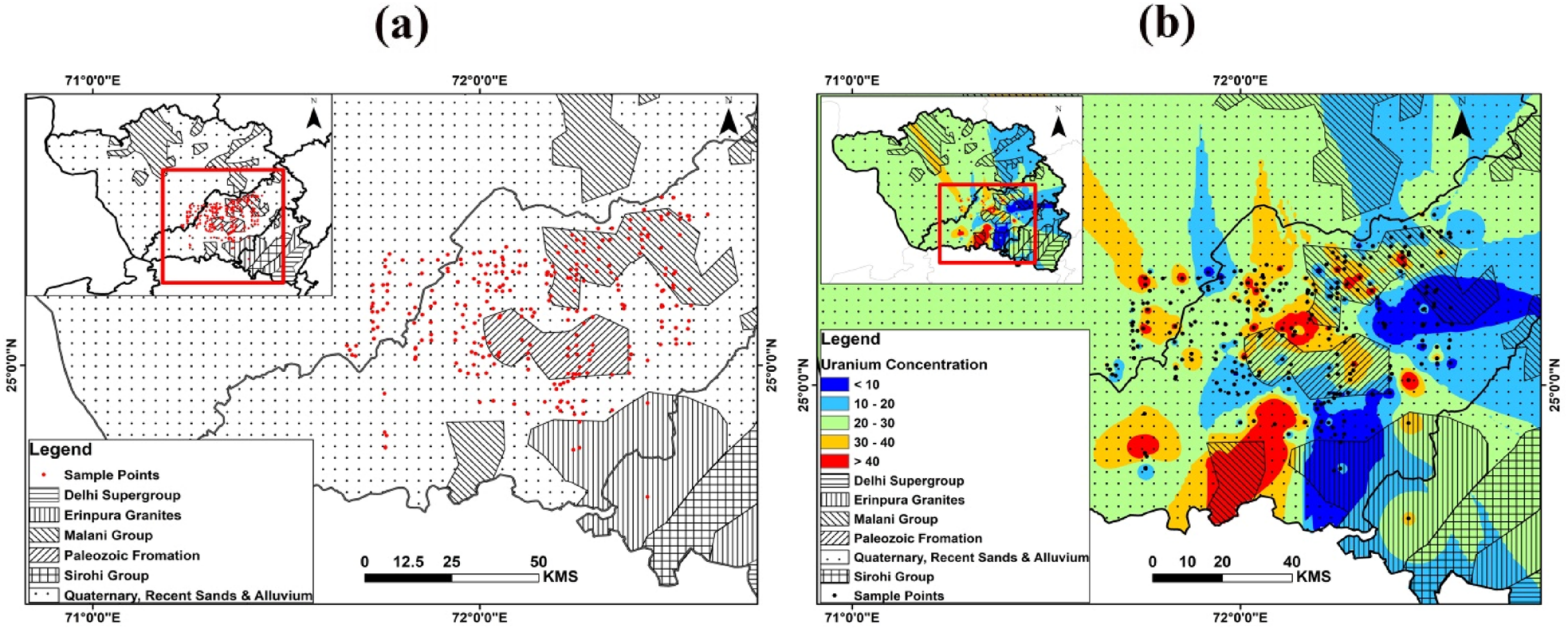
(**a**) Sampling location of the study area. The area scanned for the groundwater is in Jalore, Barmer and Sirohi districts. (**b**) Spatial distribution of uranium in the study area.

The total area was divided into a grid pattern of size 8 × 8 km^2^ to cover the entire area of each district. The sampling location and places of U occurrences are shown in Fig. 1a. During the process of investigation, 265 samples were collected from the channels making the sample density of the order of one sample per square kilometre. The sample collection and storage were done according to the standard protocols described by ISO (2012). The groundwater samples were collected in plastic bottles after having been filtered through 0.45-μm millipore filter paper and acidified with 2-M HNO_3_ (ultrapure merck) for cation and anion analysis. All the chemicals used for analysis were of analytical grade. pH was determined by pH-meter (ELICO, India) and conductivity by conductivity-meter. The pH and EC were analysed in the field. The acid–base titration method was used to analyse bicarbonate ions (HCO_3_^−^), and sulphate ions (SO_4_^2–^) were determined by turbidimetry. Chloride ions (Cl^−^) were determined using the volumetric method. F^−^ was measured using the Fluoride electrode. The cations, calcium (Ca^2+^), and magnesium (Mg^2+^) were analysed using complexometric titration using ethylenediamine tetra acetic acid (EDTA.) The heavy metals zinc (Zn), copper (Cu), lead (Pb), lithium (Li), vanadium (V) and cobalt (Co) were detected using inductively coupled plasma atomic emission spectrometry. Three replicates were used to analyze all of the samples. As part of quality control, duplicate and standard checks were performed on every ten samples. In addition, a trace element standard reference material (SRM-1643f.) was examined. Supplementary material for methodology (Table S1a) detail the instrument's operating and optimized conditions, calibration, and QA/QC details.

The U content in the samples was measured by a LED Fluorimeter (Quantalase Enterprise Pvt. Ltd. model LF- 2). It works on the principle of measurement of fluorescence of uranyl complex in the water sample. Here a pulsed LED UV light was used to excite uranyl species at 405 nm. The detection range of the instrument is 0.5–1000 ppb, with a detection limit of 0.2 ppb. Na^+^ and K^+^ were analysed using ELICO made flame photometer (Model No. 341). The accuracy and reliability of the water analysis were checked using the charge balance approach (error calculation) as follows:

1 $${\text{Error }} = \, \left\{ {{\text{I }}\left( {\Sigma {\text{Cations }} - {\text{ Anions}}} \right){\text{ I}}/ \, \left( {\Sigma {\text{Cations }} + \, \Sigma {\text{Anions}}} \right)} \right\} \, \times { 1}00$$

The details on the software used for the study; (AQUACHEM, PHREEQC, ArcGIS, and different statistical softwares) and calculations for deriving Chemical Toxicity Risk Estimation, Radiological Risk Assessment, Age-Dependent Annual Ingestion Dose for Different Age Groups, Dose Assessment to Different Organs Using Hair Compartment Model of Uranium, Heavy Metal Toxicity, HPI Index, MTL Index and Human Health Risk Assessment are provided as supplementary material for methodology (supplementary text S1).

## Results

The present arid area is marked by carnotite mineralization where V and U are the prominent elements. Various geological factors influence the concentration of U in water. Enhanced concentration of bicarbonate in groundwater augments the formation of highly soluble uranyl carbonate complexes thereby resulting in the elevated U concentration in groundwater. U in groundwater in India is generally reported in the range of 1–100 µg L^−1^. In the Indo-Gangetic plain, U has values ranging from 0.6 to 65.3 µg L^−1^ (Amritsar), 0.12–38.8 μg L^−1^ (Pathankot) and 0.12–38.8 μg L^−1^ in Gurdaspur^24^. Kumar et al.^25^ have reported the value of U to range from 0.2 to 644 μg L^−1^ in Bhatinda (Punjab). In Rajasthan, Ganganagar district, the values of U varied from 2.5 to 171 μg L^−1^. Whereas, the values in Sikar district were between 4 and 136 μg L^−1^. Meanwhile, the occasional high occurrence of PO_4_^3−^ levels (5.90 mg L^−1^) may be related to anthropogenic activities such as agriculture activities, which can also be an additional source of U in groundwater^26^. This contamination is due to the use of phosphatic fertilizers resulting in the additional contamination of groundwaters with phosphate^27^.

The statistical parameters of the physicochemical elements, U and heavy metals and standard ranges for defining the suitability of these elements in water for drinking purposes are listed in Table 1. The U concentration ranged between 0.6 and 260 μg L^−1^ with a mean value of 24.0 μg L^−1^. U concentration in 30% of the 265 groundwater sources exceeded the provisional WHO (2012) health guidelines. Results show that 69.3% of samples have U values below 30 ppb; 27.3% of samples shows values between 30 and 60 ppb; 3% of the samples indicate values between 60 and 100 ppb. Moreover, 0.38% of samples have values greater than 100 ppb. The spatial distribution of U is shown in Fig. 1b. The highest value of U is observed in the piedmont zone of Sagi and Sukri rivers. It is worth noting that MIS suites of rocks are represented by rhyolite, volcanic agglomerate/breccia, andesite, and basalts. HCO_3_—is in moderate amount, ranging from 101 to 816 mg L^−1^. SO_4_^2−^ was increasing in the northeast (NE) direction; while K^+^, CO_3_^2−^, and PO_4_^3−^ exhibited a similar distribution pattern, having low values. Spatial distribution is essential for evaluating spatiotemporal evaluation of different important parameters^28,29^. The spatial distribution of heavy metals and trace metals is indicated in Figs. S1 and S2. The spatial distribution of metals, trace elements and their origin is given in supplementary text l S3.

**Table 1:** Descriptive statistical analysis of Physico-chemical and metalloids in groundwater from western part of Rajasthan and northern part of Gujarat (n = 265).

Parameters	Min	Max	Mean	S.D	Median	25 percentile	75 percentile	Skewness	Kurtosis	Geom. mean	C.V	Stanadard guidelines in μg L^−1^ BIS Limit (IS 10,500:2012)			WHO Limit^30^	Samples
												(DesirableLimit)	(Permissible Limit)	Samples		
														Below permissible limit		Below Permissible limit
pH	6.9	9.2	8.16	0.03	8.2	7.8	8.5	− 0.02	− 0.66	8.14	5.98	6.5–8.5	6.5–8.5	71%	6.5–8.5	71%
Eh (mV**)**	407.1	542.8	483.28	28.20	483.8	460.2	507.4	− 0.06191	− 0.5422	−	0.05422					
Cond. (mho/cm)	0.39	9.48	2.87	0.1	2.575	1.6	3.9175	1.03	1.42	2.35	60.21					
TDS (mg/l)	226	5212	1571	56	1377	884.25	2078.5	1.07	1.42	1288.04	60.09	500	2000	56%	1000	32%
Na (mg/l)	20	1700	585.4	22.3	518.5	294.5	843.75	0.67	0.22	430.01	64.21	–	–		200	18%
K (mg/l)	1	64	3.75	0.31	2	2	4	7.05	69.67	2.65	138	–	10	95%	200	100%
Ca (mg/l)	10	369	88.6	4.75	67	30.25	115	1.63	2.44	59.69	90.4	75	200	93%	100	76%
Mg (mg/l)	10	314	49.56	2.69	38	18	68	2.36	7.96	35.18	91.56	30	100	90%	50	65%
Cl (mg/l)	37	2868	807.9	37.1	668.5	248.25	1283.25	0.75	− 0.1	540.09	77.42	250	1000	69%	250	28%
HCO_3_ (mg/l)	49	824	387.2	9.85	366	268	500	0.39	− 0.41	347.86	42.89					
SO_4_ (mg/l)	10	1240	206.5	13.4	139	42.5	280	1.97	4.23	112.81	109.4	200	400	89%	250	76%
PO_4_ (mg/l)	0.5	6.6	1.27	1.14	0.7	0.5	1.675	2.061	4.674	0.96	0.898					
F (mg/l)	0.7	9.4	2.67	0.09	2.3	1.5	3.7	1.17	1.57	2.28	57.68	1	1.5	26%	–	
V(µg/l)	2	156	29.76	1.4	23	17	32	2.71	8.91	23.84	79.1	–	–		–	
Li (µg/l)	10	221	89.75	2.36	90	57	115	0.44	0.2	79.69	44.39	–	–		–	
Co (µg/l)	2	47	12.77	0.49	11	6	15	1.45	2.47	10.44	65.2	–	–		–	
Ni (µg/l)	6	122	30.24	0.86	27	22	34	2.38	9.83	27.56	47.85	20	No relaxation		–	
Cu (µg/l)	4	41	9.53	0.39	6	5	12	1.68	2.61	7.96	69.01	50	1500	100	1500	100%
Zn (µg/l)	5	368	18.01	1.76	11	10	18	9.18	96.21	13.53	164.6	5000	15,000	63%	3000	100%
Pb (µg/l)	3	150	15.9	1.18	10	9	15	4.68	24.32	11.93	124.7	50	No relaxation		10	0.25
U (µg/l)	0.6	260	23.04	1.26	19	10	31	5.23	51.74	16.52	92.5					

The piper plots^31^ have been classified based on TDS values. Fig. S3 indicates the piper plots of (a) freshwater,(TDS1; TDS < 1000 mg L^−1^), (b) brackish water (TDS2; 1000 < TDS < 2000 mg L^−1^) and (c) saline water (TDS3; TDS > 2000 mg L^−1^). Most of the high U samples belong to TDS 3 group.

The higher salinity water is identified to have a high U concentration in the groundwater. Saline influence in the samples is calculated using the base exchange index (BEX). BEX can be used to identify refreshing or seawater mixing processes.

2 $${\text{BEX }} = {\text{ Na}} + {\text{K}} + {\text{Mg}} - {1}.0{\text{716Cl}} - \left( {{\text{meqL}}^{{ - {1}}} } \right)$$

A BEX around zero indicates freshwater; while a negative BEX points towards saltwater intrusion; and alternatively, a positive value refers to refreshing. The BEX index is found to be negative in only 23% of the samples.

**Figure 2:**
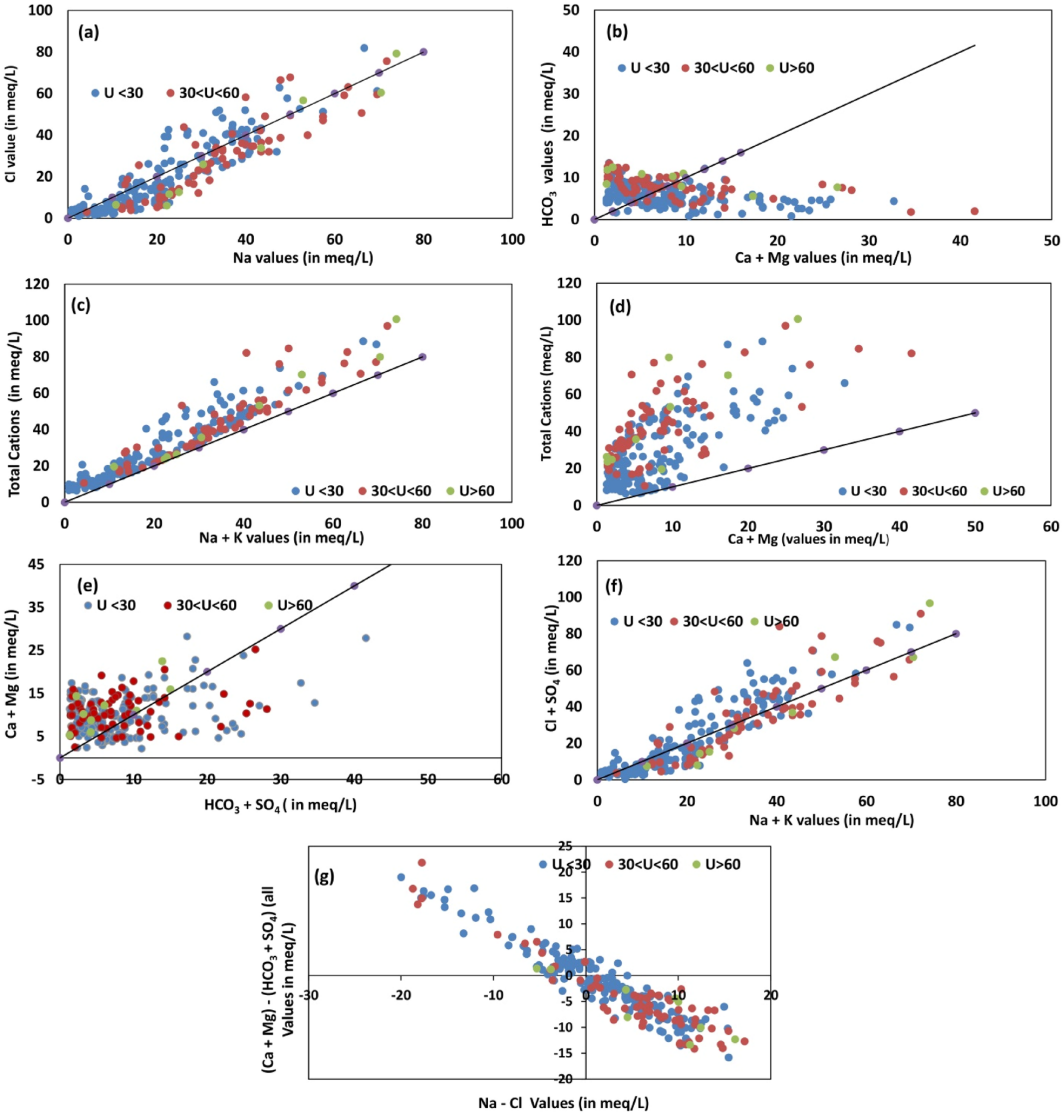
Weathering plots for (**a**) Na^+^ versus Cl^−^, (**b**) Ca^2+^ + Mg^2+^ versus HCO_3_^−^, (**c**) (Na^+^  + K^+^) versus TZ^+^ , (**d**) (Ca^2+^  + Mg^2+^) versus TZ^+^ , (**e**) (SO_4_ + HCO_3_) versus (Ca^2+^ + Mg^2+^), (**f**) (Na^+^  + K^+^) versus (Cl^−^ + SO_4_^2^ ), (**g**) (Na^+^-Cl^−^) versus (Ca^2+^ + Mg^2+^)-(SO_4_^2−+^HCO_3_^−^).

Weathering plots are used to estimate the relative contribution of geogenic source (evaporite dissolution, silicate weathering and carbonate dissolution) to its ionic load^32^, (supplementary text S3). Figure 2a–g reveal the weathering plots for Na^+^ versus Cl^−^, Ca^2+^ + Mg^2+^ versus HCO_3_^−^, (Na^+^  + K^+^) versus TZ^+^, (Ca^2+^  + Mg^2+^) versus TZ^+^, (SO_4_ + HCO_3_) versus (Ca^2+^ + Mg^2+^; (Na^+^  + K^+^) versus (Cl^−^ + SO_4_^2^) and (Na^+^-Cl^−^) versus (Ca^2+^ + Mg^2+^)-(SO_4_^2−+^HCO_3_^−^)^33^.

Principal component analysis (PCA) of the data makes it evident that water–rock interaction has played a major role in determining the hydrochemical characteristics of groundwater in the arid zone. PCA was performed as the initial step for the cluster analysis (CA) procedure. The Kaiser–Meyer–Olkin value (0.65) and the results of Bartlett’s test of sphericity (*p* < 0.001) showed that PCA could be used for data analysis. Four principal components were derived based on the Kaiser criteria of eigenvalue > 1. The percentage of variance for the four components corresponded to 25.66, 10.60, 7.95, and 7.51%, respectively.

The equilibrium state of the water concerning the mineral phase can be determined by calculating the Saturation Indices SI using analytical data. The SI of a mineral could be obtained from the following equation.

3 $${\text{SI}} = \frac{IAP}{K}_{S}$$

When SI < 1, minerals tend to dissolve; alternatively, at SI > 1, they tend to precipitate. SI revealed that saturation indices of the groundwater are supersaturated concerning some minerals like magnesite, hydroxyapatite, dolomite, apatite, aragonite, huntite, and calcite and would precipitate in optimum circumstances. The states of saturation of U minerals are found varying between 12 and 1.3, − 10 to 0.3, − 8 to − 3, − 13 to − 6 and − 17 to − 9 for schoepite, carnotite, uraninite, rutherfordine, and autunite respectively.

The mole concentration of different carbonate, sulphate, and oxide species of U was calculated by using PHREEQCI ver.3.0^34^. The carbonate species of the U mineral were UO_2_CO_3_, UO_2_(CO_3_)_2_^2−^, and UO_2_(CO_3_)_3_^4−^. The molal concentration of these species varied between 2.7 × 10^–18^ and 2.2 × 10^–11^, 7.5 × 10^–18^ to 2.3 × 10^–8^ and 3.3 × 10^–19^ to 5.5 × 10^–7^, respectively. The molal concentration of UO_2_SO_4_ ranges between 3 × 10^–25^ and 7 × 10^–18^, and that of UO_2_(SO_4_)_2_^2−^ varied between 5 × 10^–23^ and 1.6 × 10^–16^ mol.

## Discussion

### Hydrochemical facies and water type

The piper plot in Fig. S3 highlights the hydrochemical facies of U < 30 ppb, 30 ppb < U < 60 ppb and U > 60 ppb in the group TDS1, TDS2, and TDS3. The correlation analysis indicated a strong correlation between Na, Cl, EC, and TDS reflecting saline water impact. Consequently, groundwater salinity may have originated from water–rock interaction, seawater intrusion, evaporation, deposition, and fossil saline water^35^. The major hydrochemical facies observed in the different TDS group samples are given in supplementary text S3. It is observed that most of the high U content samples belonged to Na-Cl hydrochemical facies.

### Principal component analysis

The principal component 1 (PC1) showed loadings of conductivity, TDS, Na^+^, Ca^2+^ Mg^2+^, Cl^−^, U and SO_4_^2−^ contributing to 25.66% of the variance. Such grouping indicates water–rock interaction through silicate weathering and evaporite dissolution contributing to Na^+^ and Cl^−^ (Table S3). Rock source deduction revealed carbonate weathering due to the dissolution of calcite and dolomite minerals leading to the increase in pH and TDS in groundwater. As observed by Prasanna et al.^36^, the clay lens enhances ionic concentration in the adjacent groundwaters due to the process of ion exchange. The saline groundwater with the association of Ca-Mg-SO_4_ was found to facilitate the U mobilization. Further, the redox conditions due to salinity variations also promote the enrichment of U in groundwater. The long residence time of groundwater augments the salinity, and high salinity generally favours the release of U due to the increase in residence time. Principal component 2 (PC2) showed an association between HCO_3_^−^, V and U (Table S3), showing bicarbonate complexation and carnotite dissolution, leading to uranium enrichment. Carnotite is a secondary U mineral observed in calcrete deposits. Carnotite is generally reported in a region with U-enriched groundwater, along with the SO_4_^2−^ and CO_3_^2−^ facies formation, especially in arid to semi-arid climates^27^. Other factors governing the carnotite formation process are pH, Eh, pCO_2_, availability of HCO_3_, U, K etc., in groundwater, or the rate of groundwater flow, alteration process, and stagnation of water, either by chemical or physical process. Groundwater is characterised by the dissolution of carnotite predominantly containing U, V, and K^+^ (Table S3). As groundwater flows through a calcrete formation, it dissolves HCO_3_ and adjoining clays exchange K^+^ for hydrogen which leads to the increase of HCO_3_^−^ and decrease of K and pH^27^. The dissolution of carnotite in the presence of CO_2_ and water leads to the formation of K, U, V, and HCO_3_^−^

4 $${\text{KUO}}_{{2}} {\text{VO}}_{{4}} + {\text{ H}}_{{2}} {\text{O }} + {\text{ CO}}_{{2}} \leftrightarrow {\text{ K }} + {\text{ UO}}_{{2}} + {\text{ HVO}}_{{4}} + {\text{ HCO}}_{{3}}$$

Association of Pb, Ca^2+^ and K^+^ in the PCA 3, along with the negative correlation of pH represented 7.95% of the variance in PCA 3. The K^+^ in the aquatic medium may be exchanged for H^+^ in water, indicating the ion exchange process. The negative correlation of pH revealed that the cation exchange is the predominant process. PCA 4 explaining 7.51% of the variance indicated a positive correlation with heavy metals F^−^ and Ni and a negative correlation of Li and Pb. The positive association between Ni and F^−^ corresponded to their geogenic occurrence. Thus, PCA indicated that carnotite dissolution and rock water interaction including the evaporite dissolution are the processes responsible for the presence of U. Carnotite formation and speciation of U got augmented due to the leaching by meteoric waters from uranium-rich igneous rocks of the MIS. During their migration, the waters become more alkaline and saline due to progressive interactions and evaporation. Groundwater is intercepted by basement lithology and is directed towards the surface through soil suction/capillary rise where ion exchange and evaporation increases, CO_2_ degassing- pH decreases and change in water salinity, resulting in the breaking down of uranyl carbonate ions^20^. Uranyl carbonate is extremely stable in aqueous solutions, but it destabilizes due to evaporation. Decomplexation of uranyl carbonate in waters of higher salinities and evaporation converts into uranyl ion, which forms as nucleating seed over clay or hydroxide of iron and aluminium^37^. Subsequently, in the presence of V and K^+^ ions, carnotite precipitation takes place in the pH range of 6.0–8.0. Given that higher V in groundwater is rare, V may be found present in alkali water of U-V mineralized regions. The higher V prohibits the movement of U^6+^ in the pH between 4.0 and 8.0, leading to the thermodynamic stabilization of carnotite.

**Figure 3:**
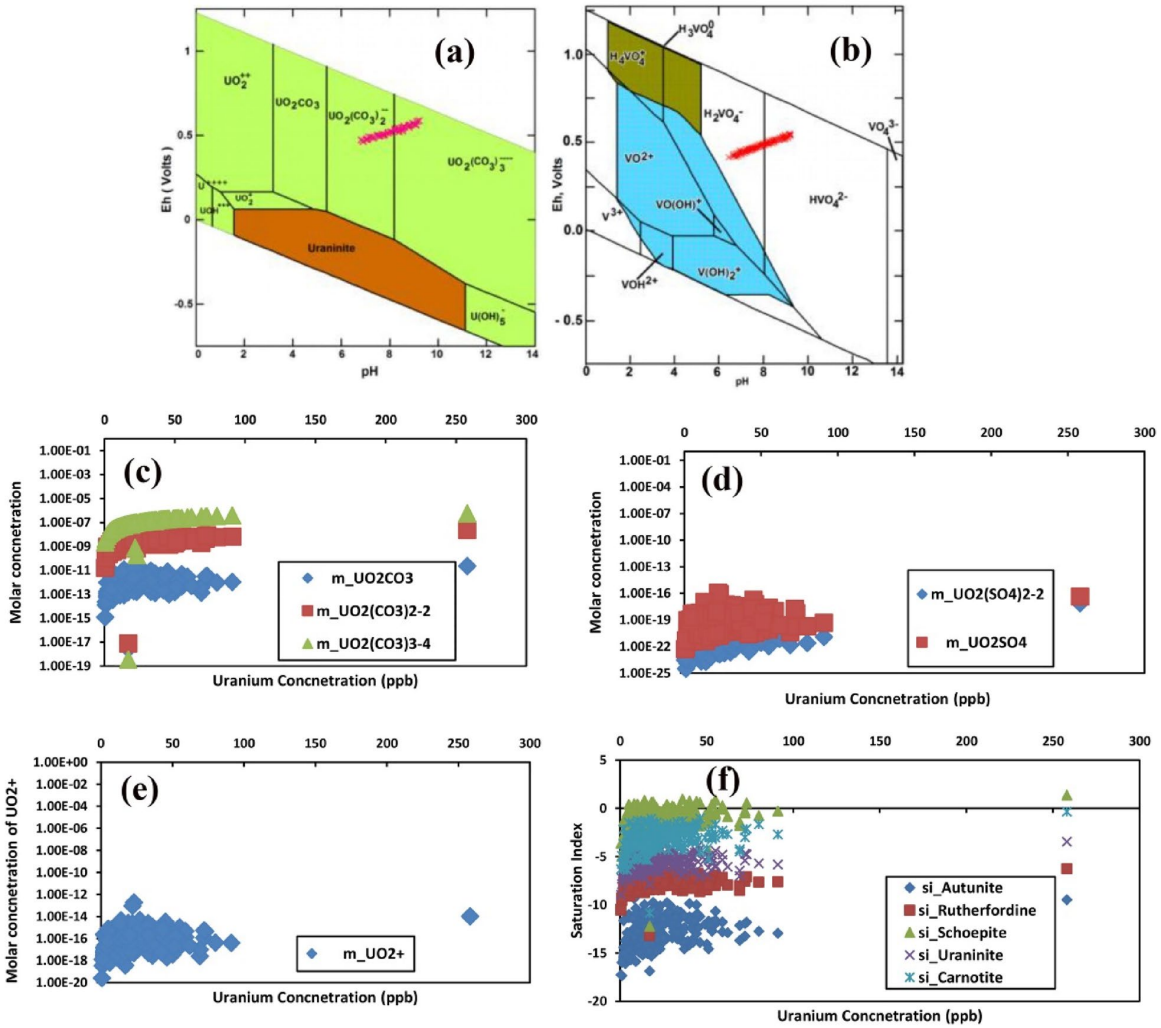
(**a**) The stability states of different uranyl entities, the green shades indicate the species and the brown represents the mineral and (**b**) Vandyl species, the lighter and the darker shades indicate the fields of V^4+^ and V^5+^ complexes, in the Eh–pH plots. Variation of the (**c**) molar concentration of uranyl carbonates species (**d**) Uranyl sulfate. species. (**e**) Uranyl oxide, with U concentration in groundwater. The species concentrations were derived from the PHREEQCI. (**f**) The relationship of the saturation index of major uranium minerals with Uranium concentration in groundwater.

The predominant U species in groundwater was calculated by Eh–pH diagram. Stanley and Wilkin^38^ argued that Eh–pH diagram constructed in the presence of hydroxide, sulphate, and carbonate ligands showed that calcrete waters were mostly characterised by neutral to alkaline and oxidising conditions favouring the U mobility through the formation of soluble anionic carbonate complexes, such as UO_2_(CO_3_)_2_^2−^. Moreover, it is known that the mobility and absorption by phosphate, clay minerals organic materials, and iron oxides in an oxidising environment are governed by Eh and pH, While the adsorption dominates at low pH, the higher pH favours the formation of anionic uranyl carbonates. As early as 1978, Langmuir^39^ described uranyl complexation and attributed it to pH and Eh as the governing factors of the process. In groundwater with higher Eh (oxidizing) and lesser pH (~ 5), the major forms of U are the uranyl ion UO_2_^2+^ as U^6+^. In the presence of F^−^, these waters lead to the formation of uranofluoride complexes. Subsequently, when the pH increases in these oxidising waters, the uranyl ion tries to accommodate the CO_3_^2−^ to form the uranyl-carbonate complexes as UO_2_(CO_3_)_2_^2−^. Correspondingly, at higher pH, it forms UO_2_(CO_3_)_3_^4−^^40^. The Eh–pH diagram for uranyl species (Fig. 3a) shows that the pH ranges from 6.9 to 9.2, and Eh ranges from 407 to 542 mV. The diagram shows that stability ranges for different forms of U concerning CO_3_^2−^ and hydroxyl ion association with U, like U^4+^, UOH^3+^, UO_2_^+^, Uraninite and U (OH)^−5^. The increase in pH shows the variation of uranyl form from UO_2_ to UO_2_CO_3_, UO_2_(CO_3_)_2_^2−^, UO_2_(CO_3_)_3_^4−^. The availability of oxygen increases with Eh, subsequently, CO_3_^2−^ increasing with pH. The samples in the UO_2_(CO_3_)_2_^2−^ and UO_2_(CO_3_)_3_^4−^ fields, reflecting the availability of CO_3_^2−^ ions, and thus, increases uranyl carbonate species. The Eh–pH diagram of vanadium revealed the speciation of V. V mobility in natural waters mainly depends on its ability to form anion complexes. In the present study, HVO_4_^2−^ and H_2_VO_4_^−^ are the predominant V^5+^ species in groundwater at pH ranges between 4.0 and 9.0 and indicates an oxidizing environment. The development of thermodynamic stable complexes of certain ligands with Vanadyl ions shows the persistence of species at pH > 6 (Breit and Wanty 1991) (Fig. 3b).

The order of dominance concerning the concentration of uranium carbonate species showed the trend UO_2_CO_3_ < UO_2_(CO_3_)_2_^2−^ < UO_2_(CO_3_)_3_^4−^ reflecting the enhanced availability of carbonate ions (Fig. 3c). A parallel increase of CO_3_^2−^ became evident concerning U ions in groundwater, leading to the complexation of uranyl-carbonates. The concentration of uranyl sulphate species UO_2_(SO_4_)_2_^2−^ and UO_2_SO_4_ were compared in groundwater samples (Fig. 3d). The ranges of sulphate species with U showed UO_2_SO_4_ with a higher concentration than UO_2_(SO_4_)_2_^2−^. While these species showed an increase in the concentration of these compounds. However, the samples are found to have been dispersed and not found to reflect a definite linear trend. The uranyl oxide (UO_2_^+^) (Fig. 3e) also reflected a similar trend, ranging between 2.6 × 10^–20^ and 9.3 × 10^–14^ mol.

### Saturation indices and water mineral equilibrium

The supersaturation of groundwater concerning calcium and magnesium bearing minerals suggested that there is a depletion of calcium and magnesium content due to their mineral precipitation in the groundwater system. In contrast, the samples were undersaturated with respect to evaporite minerals, such as anhydride, artinite, brucite, epsomite, fluorite, gypsum, and halite. These results indicate that the mineralization is linked to the dissolution of evaporitic minerals (halite, anhydrite, and gypsum). Saturation states of chief uranium minerals, such as schoepite (UO_2_(OH)_2_.H_2_O), carnotite (KUO_2_VO_4_), uraninite (UO_2_), rutherfordine (UO_2_CO_3_), and autunite Ca(UO_2_)_2_(PO_4_)_2_ are shown in Fig. 3f. The thermodynamic state of groundwater showed saturation to near saturation for the composition of schoepite and under saturation to near saturation for carnotite. Other mineral compositions also reported under saturation, revealing a tendency to dissolve. The saturation states of the uranium minerals are in the following order, namely, schoepite > carnotite > uraninite > rutherfordine > autunite. Several mechanisms, including sorption, colloidal precipitation, change in vanadium redox state and CO_2_ partial pressure (pCO2), and pH have been proposed to explain the precipitation of carnotite. The corresponding carnotite formula is given in supplementary information (**S3**).

All these minerals are complexes of UO_2_ (Uraninite). The association of this complex with the hydroxyl molecule leads to schoepite, and with carbonates, it leads to the saturation of rutherfordine. The Ca(PO_4_)_2_ and KVO_4_ association results in saturation states of autunite and carnotite, respectively. The calcrete observed inland could be mainly due to the carbonate rock precipitation in the phreatic zone of an aquifer^41^. The presence of calcrete in this zone facilitates the interaction between the laterally moving groundwater either by ion exchange or by chemical reaction process. The regions with drastic water level fluctuation, especially in the structurally disturbed hard rock aquifers favour calcrete deposits, and if the drainage is known to be rich in U, the carnotite minerals along these planes can be expected. This generally happens when the U from the granitic formation like MIS is released and combines with the V, derived from the mafic rocks. The MIS consists of complex acidic and is intrusive. V was also observed to be in Fe silicate minerals, like biotite and amphibole as a replacement for Fe in the crystal lattice^18^. An earlier investigation showed a similar occurrence of calcrete deposits in Australia having U associated with V^42^. Again, even much earlier than the year 1999, it was reported that the higher oxidation state of V facilitates the precipitation of U from the solution. The thermodynamic state of the carnotite composition is in near saturation conditions, even while other mineral compositions like uraninite is found undersaturated Fig. 3f. The stagnated water conditions favour evaporation, change in pCO_2_ and pH, and subsequent saturation of waters with carbonate and calcium that ultimately help in the precipitation of calcium carbonates (calcrete).

### Health risk assessment due to uranium

#### Age-dependent annual effective dose

The detailed statistical analysis results of age-adjusted and gender-specific annual ingestion dose in groundwater in arid regions are shown in Table 2. The greatest mean annual ingestion dose is observed in infants (0–6 mo) and (7–12 mo) were 49.30 and 56.34 µSvy^−1^ in comparison to all other age groups. Annual ingestion dose due to U in drinking water for males in age group 9–13, 14–18, and > 18 years ranged between 0.74 to 119.13; 1.01 to 161.40 and 0.22 to 35.11 µSvy^−1^, respectively. For females, it varied between 0.65 to 104.24; 0.70 to 112.49 and 0.16 to 25.62 µSvy^−1^ in the same order of age groups (Table 2). The annual effective dose due to the ingestion of U was found to be less than 100 µSvy^−1^ as per the European Commission (Table 2). Thus, the values are found within the radiological risk.

**Table 2:** Radiotoxicity and chemical toxicity due to U (here U-238, U-235 and U-234 are isotopes of Uranium) in groundwater (DWI = Daily water intake).

Categories		DWI	Min	Max	Median	Mean	First Quartile	Third Quartile	IQR
**Annual ingestion dose due to uranium intake (µSvy-1)**									
Infant	Age: 0–6 months	0.7	1.086	173.74	41.263	49.305	21.718	69.496	47.779
	Age:7–12 months	0.8	1.241	198.56	47.158	56.349	24.82	79.424	54.604
Children	Age:1–3 years	1.7	0.712	113.88	27.047	32.318	14.235	45.552	31.317
	Age:4–8 years	2.4	0.621	99.28	23.579	28.175	12.41	39.712	27.302
Male	Age:9–13 years	2.4	0.745	119.136	28.295	33.809	14.892	47.654	32.762
	Age:14–18 years	3.3	1.009	161.403	38.333	45.804	20.175	64.561	44.386
	> 18	3.7	0.219	35.113	8.339	9.965	4.389	14.045	9.656
Female	Age:9–13 years	2.1	0.652	104.244	24.758	29.583	13.031	41.698	28.667
	Age:14–18 years	2.3	0.703	112.493	26.717	31.924	14.062	44.997	30.936
	Age: > 18	2.7	0.16	25.623	6.085	7.272	3.203	10.249	7.046
	Pregnancy	3	0.178	28.47	6.762	8.079	3.559	11.388	7.829
	Lactation	3.8	0.225	36.062	8.565	10.234	4.508	14.425	9.917
**Analysis of Uranium concentration and asociated toxicity of ground water samples from Jalore, Barmer and Sirohi District**									
Uranium Conc. (µg L^−1 )^			0.6	260	19	23.12	10	31	21
UraniumActivity (Bq L-1)			0.013	6.45	0.48	0.58	0.25	0.78	0.52
Cancer		U-238	9.37 × 10^–13^	4.84 × 10^–12^	3.56 × 10^–11^	4.33 × 10^–11^	1.87 × 10^–11^	1.81 × 10^–11^	3.93 × 10^–11^
Mortality		U-235	7.75 × 10^–13^	2.26 × 10^–9^	1.66 × 10^–10^	2.09 × 10^–10^	1.55 × 10^–11^	4.98 × 10^–11^	3.41 × 10^–11^
Risk (Bq-1)		U-234	7.62 × 10^–13^	3.93 × 10^–10^	2.89 × 10^–11^	3.52 × 10^–11^	1.53 × 10^–11^	4.72 × 10^–11^	3.20 × 10^–11^
Cancer		U-238	9.37 × 10^–12^	4.84 × 10^–9^	3.56 × 10^–10^	4.34 × 10^–10^	1.87 × 10^–10^	5.81 × 10^–10^	3.93 × 10^–10^
Morbidity		U-235	1.25 × 10^–12^	6.37 × 10^–10^	4.69 × 10^–11^	5.71 × 10^–11^	2.47 × 10^–11^	7.65 × 10^–11^	5.18 × 10^–11^
Risk (Bq-1)		U-234	1.18 × 10^–12^	6.12 × 10^–10^	4.51 × 10^–11^	5.66 × 10^–11^	2.37 × 10^–11^	7.36 × 10^–11^	4.98 × 10^–11^
LADD (µg kg^−1^ d^−1)^			0.011	5.24	0.39	0.47	0.2	0.63	0.42
HQ			0.017	8.74	0.64	0.78	0.339	1.05	0.71

#### Radiological and chemical toxicity risk

Radiological risk assessment due to U ingestion was calculated by estimating the cancer risk. Table 2. There are three naturally occurring isotopes of U with half-lives of 2.4 × 10^5^, 7.0 × 10^8^, and 4.5 × 10 ^9^ years. The cancer mortality risk coefficients Bq L^−1^ for the three isotopes of U ^234^U, ^235^U, ^238^U were taken to be 6.1 × 10 ^−11^, 6.2 × 10^–11^, and 7.5 × 10 ^−10^, respectively. The average values for cancer mortality risk by U isotopes ^234^U, ^235^U, and ^238^U are found 4.3 × 10^–11^, 2.09 × 10^–10^, and 3.52 × 10^–11^, respectively. Similarly, the mean cancer morbidity risk for ^238^U, ^235^U, and ^234^U are 4.34 × 10^–10^, 5.71 × 10^–11^, and 5.66 × 10^–11^, respectively. U is a nephrotoxin that may result in kidney damage. Similarly, the chemotoxic dose was also computed for estimating health hazards. The lifetime average daily dose (LADD) found with a minimum and maximum value between 0.011 and 5.24 µg kg^−1^ d^−1^, with a mean value of 0.47 µg kg^−1^ d^−1^, which is much less than the reference dose (RfD) of 0.6 and 4.4 µg kg^−1^d^−1^ prescribed by WHO^43^ and AERB^12^, respectively. Also, the hazard quotient (HQ) shows arithmetic mean (AM), median and inter-quartile range (IQR) of 0.78, 0.64, and 0.71, respectively. Since HQ is less than unity, the analysed water samples could be used for drinking thus showing no considerable radiation risk to the population of the area under investigation (Table 2). Doses to various organs/tissues of an adult human are summarized in Table S4. Po-210 was seen to have been the major contributor to the dose to all organs, except for bone surface, for which Pb-210 is the source. The annual effective ingestion dose to the whole body was observed to have spanned between 0.114 and 41.791 µSv with an average of 4.710 µSv, which is of much less value than the recommended limit of 100 µSv^43^. Bone surfaces has the maximum share of dose (38%) due to U and its daughters, followed by kidneys (14%), LLI Wall (12%), liver (5%), ULI Wall (5%), and small red marrow (4%). The dose coefficients calculated with the hair model were seen to be lower than ICRP’s^44^ biokinetic model because a considerable fraction of U in the blood (about 31.5%) is known to have excreted into the hair, and therefore resulting in no contribution to dose**.**

This study elaborates the behaviour of uranium in groundwaters of complex terrain, especially in arid regions. The association of U to major ions and metals helps to understand the nature of reactions in groundwater of similar climatic conditions. The health risk assessment and identification of regions with higher U in groundwater helps the policymakers to manage the utility of the available water resources.

#### Classification of groundwater-based heavy metal index

The metal toxicity load (MTL) index was developed to find the toxicity of various metals in the groundwater samples (Table 3). From the results, it was found that 18.67% of sampling sites showed that toxicity of Ni was below their permissible toxicity load (825 mg L^−1^); while 81.33% of sites exceeded their permissible toxicity load. Similarly, 1.05% sampling sites exceeded the toxicity of Zn for permissible toxicity load, which is 136.95 mg L^−1^. The 3.87% sampling sites went above toxicities of Pb for permissible toxicity load, which is 76.55 mg L^−1^; 27.11% sampling sites showed to have gone above toxicities of U for permissible toxicity load, which is 24.99 mg/L. Their percentage removal varied from 3.22% to 88.37%. Nonetheless, for F, V, Co, Cu, and Pb, their toxicity range showed to be below the permissible toxicity load. On the whole, the range of toxicity for metals was found above the permissible toxicity load (315.19). The heavy metal pollution index (HPI) index was calculated for the metals of groundwater (Table 3). From the results of HPI, it was observed that HPI value was found to be less than 100, signifying less pollution in the study area.

**Table 3:** Metal toxicity load of the groundwater following ATSDR (2017) relative to the toxicity level of metals accountable for human beings and computation of heavy metal pollution index (HPI) for groundwater of western Rajasthan and Northern Gujarat.

Metals	Mean (µg/L)	Hazard intensity score (HIS)	Permissible toxicity load (mg L^−1^)	Range of toxicity of heavy metals (mg L^−1^)	Highest permitted value for drinking water (S_i_)	Maximum desirable value (I_i_)	Sub-index (Q_i_)	Unit weight Wi = (K/S_i_)	W_i_ × Q_i_
F	2.67	550	825	0.38–5.17	1000	1500	299.46	0.001	0.299
V	29.76	648	129.6	1.29–101.08	200	–	14.88	0.005	0.074
Li	89.75				700	–	12.82	0.0014	0.0183
Co	12.77	1011	101.1	2.02–47.51	100	40	− 45.38	0.01	− 0.453
Ni	30.24	993	19.86	5.95–121.14	3000	–	1	0.00034	0.00034
Cu	9.53	805	1207.5	3.22–33.00	1500	50	− 2.79	0.00067	− 0.0018
Zn	18.01	913	136.95	4.56–335.98	15,000	5000	− 49.81	6.67E−05	− 0.0034
Pb	15.9	1531	76.55	4.59–229.65	50	–	31.8	0.02	0.636
U	23.04	833	24.99	0.41–214.91	30	–	76.8	0.034	2.56
Range of metal toxicity load (MTL) (mg/L) = 63.13–427.82					∑Wi = 0.0718; ∑ Wi × Qi = 4.046 and HPI = 56.34				

#### Health risk assessment due to heavy metals

To understand the non-carcinogenic risk associated with heavy metals, an average daily dose of exposure CDI_ing_ and CDI_derm_, Hazard Quotients, $HQ_{ing}$ and $H_{Qderm,}$ and Hazard Index (HI) corresponding to ingestion and dermal pathways were calculated separately for adults and children as tabulated in Table 4. The maximum daily dose in case of adults belonged to Li, i.e., 2.139 mg/kg/d in drinking water. The CDI_ing_ in the case of adults followed the order Li (2.139) > V (0.777) > Ni (0.761) > Pb (0.594) > Zn (0.466) > Cu (0.332) > Co (0.319) mg/kg/d. In the case of children, the CD_ing_ followed the trend Li (3.195) > V (1.161) > Ni (1.137) > Pb (0.887) > Zn (0.696) > Cu (0.496) > Co (0.476). The $CDI_{derm}$ adult magnitudes followed the sequence Li > V > Pb > Cu > Ni > Co > Zn. The $CDI_{derm}$ child magnitudes followed the sequence Li (3.29 × 10^–2^) > V (1.2 × 10^–2^) > Pb (9.2 × 10^–3^) > Cu (5.1 × 10^–3^) > Ni (2.3 × 10^–3^) > Co (2.0 × 10^–3^) > Zn (7.0 × 10^–4^) mg/kg/d. The HQ values determined for adults were in the order of Li (5.093) > Co (1.062) > Pb (0.4245) > V (0.110) > Ni (3.8 × 10^–2^) > Cu (8.3 × 10^–3^) > Zn (1.6 × 10^–3^). The HQ_ing_ children followed the trend Li > Co > Pb > V > Ni > Cu > Zn. In the case of both adults and children, the HQs of Li contributed predominantly to the integrated HQ values due to higher concentrations among the metals considered. Since the average HI value for adults (0.965) showed to be less than unity and for the children (1.446) to be greater than unity; consequently, a significant non-carcinogenic risk was diagnosed for children. The mean carcinogenic risk was observed to be 7.62 for children, conversely, 5.08 for adults. The cancer index values for Ni and Pb were found higher than the ranges recommended by USEPA^45^ of 1 × 10^–6^ and 1 × 10^–4^ for both adults and children (Table 4).

**Table 4:** Carcinogenic and non-carcinogenic health risk assessment of heavy metals in groundwater for children and adults.

Heavy metals	Children							Adults						
	Non-carcinogenic risk				Carcinogenic risk			Non-carcinogenic risk				Carcinogenic risk		
	CDI _Ing_ (Child)	CDI _Der_ (Child)	HQ_ing_ (Child)	HQ_der_ (Child)	CR _Ing_ (Child)	CR _Der_ (Child)	CI (Child)	CDI _Ing_ (Adult)	CDI _Der_ (Adult)	HQ _ing_ (Adult)	HQ _Der_ (Adult)	CR _Ing_ (Adult)	CR _Der_ (Adult)	CI (Adult)
Pb	0.887	0.0092	0.633	0.0065	7.54	0.08	7.62	0.594	0.0031	0.4245	0.0022	5.05	0.03	5.08
Cu	0.496	0.0051	0.0112	0.0006	–	–	–	0.332	0.0017	0.0083	0.00022	–	–	
Zn	0.696	0.0007	0.0023	0.000012	–	–	–	0.466	0.0002	0.0016	0.000004	–	–	–
Ni	1.137	0.0023	0.0568	0.0029	0.96	0.0007	0.96	0.761	0.0008	0.038	0.001	0.64	0.001	0.64
Co	0.476	0.002	1.586	0.0327	–	–	–	0.319	0.0007	1.062	0.0111	–	–	–
V	1.161	0.012	0.166	0.0017	–	–	–	0.777	0.0041	0.11	0.0006	–	–	–
Li	3.195	0.0329	7.606	0.0784	–	–	–	2.139	0.0111	5.093	0.0265	–	–	–

*CDI* chronic daily intake, *Ing* ingestion, *Der* dermal, *HQ* hazard quotient, *CR* carcinogenic risk.

## Conclusion

Hydrogeochemistry and geochemical speciation is carried out to investigate the mechanism responsible for the enrichment of U in the calcretes of the arid region of western Rajasthan. The non-carcinogenic and carcinogenic risks of the trace metals and uranium in the groundwater were also evaluated for health risk assessment. Carnotite dissolution is identified to be the primary cause of U enrichment in calcrete waters. High U concentration in groundwater presented a distinctive hydrogeochemical characteristic as follows: high TDS and alkalinity along with high Na^+^ and K^+^ concentration, suggesting that weathering of geogenic source material, evaporation, and dissolution from mineral surfaces are the principal mechanism of U release. Based on the TDS values, the groundwater samples were divided into TDS < 1000 mg L^−1^ (Ca-MgHCO_3_ type), 1000 < TDS < 2000 mg L^−1^ and TDS > 2000 mg L^−1^ (Na-Cl) type of water. High evaporation rates, typical of arid and semiarid climates generate saline groundwaters and alkaline pH, releasing U and F^−^ from both the primary and the secondary material sources. Despite being a U-mineralized area, only 30% of samples exceeded their recommended WHO, since carnotite was seen to have been least affected by the dissolution in the low pCO_2_ in desert soils and groundwater due to the absence or paucity of organic activity in the soil. Chemical speciation, computed using PHREEQCI of uranium/carbonate system, indicated that the predominant U species in alkaline conditions was UO_2_(CO_3_)_3_^4−^. HVO_4_^2−^ and H_2_VO_4_^−^ were the predominant V^5+^ species in groundwater in the completed study. Hydrogeochemical characteristics indicated that ionic load resulted from halite dissolution, silicate weathering, and ionic exchange. The metal toxicity load index suggested that the range were above the permissible toxicity load. The heavy metal pollution index obtained in this study is 56.34, signifying moderate pollution of heavy metals in the groundwater quality of the study area. The non-carcinogenic risks exhibited that Li, V, and Ni are the key pollutants affecting the health of human beings; conversely, carcinogenic risks inferred that Pb showed high health risks. However, the concentration of V, Li, Co, Cu, Zn, and Pb were found to be lower than those recommended by BIS^13^. 20% of samples surpassed their Ni limits. The aforesaid findings confirm the crucial role of hydrogeochemical processes in heavy metal enrichment in calcrete water. The results will be highly useful in understanding processes controlling heavy metal enrichment in arid waters.

## Supplementary Information

Supplementary Information 1.

Supplementary Information 2.

Supplementary Information 3.
