# Author Correction: Tracing geochemical sources and health risk assessment of uranium in groundwater of arid zone of India

**DOI:** 10.1038/s41598-022-17005-5

**Published:** 2022-07-22

**Authors:** P. Pandit, Atul Saini, Sabarathinam Chidambaram, Vinod Kumar, Banjarani Panda, A. L. Ramanathan, Netrananda Sahu, A. K. Singh, Rohit Mehra

**Affiliations:** 1 USICT, GGSIPU, Dwarka, Delhi, 110078 India; 2 Department of Geography, Delhi School of Economics, University of Delhi, Delhi, 110007 India; 3 Present Address: Delhi School of Climate Change & Sustainability, Institution of Eminence, University of Delhi, Delhi, 110007 India; 4 Water Research Center, Kuwait Institute for Scientific Research, Kuwait City, Kuwait; 5 Department of Botany, Government Degree College, Ramban, Jammu, 182144 India; 6 Water Sciences Lab, University of Nebraska-Lincoln, Lincoln, NE USA; 7 School of Environmental Sciences, Jawahar Lal Nehru University, New Delhi, 110067 India; 8 Maharaja Surajmal Institute of Technology, USICT, GGSIPU, New Delhi, 110058 India; 9 B. R. Ambedkar National Institute of Technology, Jalandhar, 144011 India

Correction to: *Scientific Reports* 10.1038/s41598-022-05770-2, published online 01 June 2022

The original version of this Article contained an error in the spelling of the author Sabarathinam Chidambaram which was incorrectly given as Sabarathinam Chidhambaram.

The original Article has been corrected.

