# Supplementary material for: Tracing geochemical sources and health risk assessment of uranium in groundwater of arid zone of India
Source: Sci Rep. 2022 Jun 1;12:2286. doi: 10.1038/s41598-022-05770-2 (PMC9160070; doi:10.1038/s41598-022-05770-2)
Supplement: Supplementary file 1 — Supplementary Information 1. [file 41598_2022_5770_MOESM1_ESM.docx]

**SUPPLEMENTARY TEXT (S1)**

**Methodology section**

**Table S1a Analytical precision For metals:**

| **Element** | **Wave length (nm)** | **Limit of Detection (µg/L)** | **Calibration Range for samples (µg/L)** | **Correlation Coefficient** | **Std check (µg/L)** | | **Standard Reference Material (SRM-1643f ) µg/L)** | |
| --- | --- | --- | --- | --- | --- | --- | --- | --- |
|  |  |  |  |  | **Std Value** | **Observed Value** | **Certified value** | **Observed value** |
| **Zn** | 213.857 | 0.1 | 5.0-640 | 0.99994 | 5 | 5.08 | 74.4 | 72.32 |
|  |  |  |  |  | 20 | 20.96 |  |  |
|  |  |  |  |  | 40 | 39.25 |  |  |
|  |  |  |  |  | 160 | 158.7 |  |  |
| **Cu** | 327.395 | 0.1 | 1.25-40 | 0.99967 | 1.25 | 1.26 | 21.66 | 21.3 |
|  |  |  |  |  | 5 | 5.12 |  |  |
| **Li** | 670.783 | 0.01 | 0.1-5.0 | 0.99976 | 1 | 1.13 | 16.59 | 16.1 |
|  |  |  |  |  | 2.5 | 2.71 |  |  |
| **Pb** | 220.353 | 1.3 | 5.0-40.0 | 0.99997 | 2.5 | 2.36 | 18.488 | 18.1 |
|  |  |  |  |  | 40 | 38.72 |  |  |
|  |  |  |  |  | 160 | 158.05 |  |  |
| **V** | 292.401 | 0.2 | 6.25-100 | 0.99998 | 5 | 5.32 | 36.07 | 34.41 |
|  |  |  |  |  | 12.5 | 12.41 |  |  |
|  |  |  |  |  | 25 | 24.33 |  |  |
| **Co** | 238.892 | 0.3 | 5.0-40.0 | 0.99987 | 2.5 | 2.4 | 25.3 | 24.57 |
|  |  |  |  |  | 10 | 9.56 |  |  |
|  |  |  |  |  | 20 | 18.92 |  |  |

**Analysis of Uranium:**

The total U concentration was determined using a U laser fluorimeter. This instrument's analytical technique can detect total U levels in aqueous medium at trace and ultra-trace concentrations. A laser pulse of 337.1 nm was used for excitation. To avoid matrix effects, the samples were analyzed using a laser uranium analyzer using the standard addition method. Standard addition was accomplished by gradually introducing increments of the standard into a single measured volume of the sample (Devaraj et al 2021).

After each addition, measurements were taken on the original sample as well as the sample plus the standard. A standard stock solution of 0.973-g/L U (Aldrich brand) was diluted for working concentrations and system calibration. Because uranyl phosphate complexes are stable, sodium pyrophosphate (5%) was used as a fluorescence enhancer and for the formation of uranyl complexes (Sahoo et al., 2009).

1. **Hydrogeochemical Processes**

The representative analysis of the groundwater solute chemistry was performed using Piper plots by utilizing AquaChem 9.0 software. Saturation index (SI) of the minerals and U speciation under the influence of redox conditions and complexant concentration in groundwater were calculated using the hydrochemistry software PHREEQC version 3.0.

1. **Environmental Implication**
   - 1. **Radiological Risk Assessment**

Radiotoxicity risk assessment due to U intake through groundwater for adults and children was performed by calculating excess cancer risk (CR) depending on the specific activity and isotopic composition of U. The corresponding formula and cancer risk coefficients are given in supplementary text S2, **Table S1.**

- - 1. **Chemical Toxicity Risk Estimation**

Uranium is nephrotoxin; and hence, the chemical toxicity was quantified in terms of LADD and HQ (Supplementary text **S2, Table S1**)**.**

- - 1. **Age-Dependent Annual Ingestion Dose for Different Age Groups**

The annual ingestion dose due to U intake via drinking water for different age groups was estimated by the equation given by the International Commission on Radiological Protection (ICRP, 2012) ( Supplementary text **S2**, **Table S1)**.

- - 1. **Dose Assessment to Different Organs Using Hair Compartment Model of Uranium**

The hair compartment model is a biokinetics model that describes the passage, absorption, and retention of U in various organs/tissues and excretion routes following chronic intake via ingestion (Li et al., 2009).

1. **Heavy Metal Toxicity**

Heavy metal pollution index (HPI) and metal toxicity load (MTL) were enumerated to find the pollution level of different heavy metals in the groundwater.

- - 1. **HPI Index**

HPI Index provides the collected influence of metals on the quality of water (Sheykhi and Moore, 2012). The rating is given to this index based on the relative importance of metal and defined as inversely proportional to the highest permitted standard value (Si) for individual metal and calculated as follows:

$\text{HPI}=\sum\frac{W_{i}Q_{i}}{W_{i}}$ (1)

Where *W_i_* and *Q_i_ are* the unit weight and sub-indices of F, V, Li, Co, Ni, Cu, Zn, Pb and U. The sub-index (*Q_i_*) is determined as follows: $\text{ }$

$Q_{i}=\sum\frac{\left| M_{i}-I_{i} \right|}{S_{i}-I_{i}}100$ (2)

where *M_i_* (µg L^-1^) is the determined value of F, V, Li, Co, Ni, Cu, Zn, Pb, and U. *S_i_* and *I_i_* are the highest permitted and maximum desirable values of metals in drinking water adapted from EPA (2009), BIS (2012), and WHO (2017) for metals (µgL^-1^). HPI values above 100 indicate detrimental impact, and water is unsafe for consumption; while HPI values below 100 signify less contamination of metals.

- - 1. **MTL Index**

MTL index appraises the metal content in water which influences the health of human beings. It is developed by multiplying the observed metal concentration with their hazard intensity:

$\text{MTL}=\sum M_{i}\times\text{HIS}_{i}$ (3)

where *HIS_i_* is the hazard intensity score of the i^th^ metal taken from ATSDR (2017). It is assigned as per the occurrence of the incidence of the metal as an injurious substance on the National Priorities List (NPL) sites sustained by the Agency for Toxic Substances and Disease Registry (ATSDR), based on their human exposure and their toxicity. The maximum HIS for metal was 1800; while 600 points were distributed for every NPL occurrence, toxicity, and human exposure.

- - 1. **Human Health Risk Assessment**

The human health risk assessment method was introduced to identify the degree of heavy metal exposure according to the toxicity and response of human health (Koki et al., 2015). The equations used to compute the degree of ingestion rate and dermal absorption of heavy metal in a human body through water according to USEPA (2004) is as follows:

$\text{CDI}_{\text{ingestion}}=\frac{C_{i}\times\text{IR}\times\text{EF}\times\text{ED}}{\text{BW}\times\text{AT}}$ (4)

$\text{CDI}_{\text{dermal}}=\frac{C_{i}\times\text{SA}\times K_{p}\times\text{ET}\times\text{EF}\times\text{ED}\times\text{CF}}{\text{BW}\times\text{AT}}$ (5)

The hazard quotient (HQ) and hazard index (HI) was enumerated as per USEPA (2013) to be as follows:

$\text{HI}=\sum\text{HQ}_{i}=\text{HQ}=\frac{\text{CDI}_{i}}{\text{RfD}_{i}}$ (6)

The corresponding parameters are given in **Table S2**.

The carcinogenic risk (CR) is defined as the incremental probability of developing cancer in human beings due to exposures to potential carcinogens. The carcinogenic risk was appraised by the equation

$\text{CI}=\sum\text{CR}_{i}=\text{CR}=\text{CDI}_{i}\times\text{SF}$ (7)

The corresponding parameters are given in Table **S2**.

1. **Statistical Analysis and Geospatial Database**

The spatial analyst module of Arc GIS 10.1 was used to generate the heavy metals and physicochemical parameter maps for the study area. To find the correlation among the physicochemical parameters, heavy metals and U, principal component analysis (PCA) and correlation analysis were performed using statistical programme for social sciences (SPSS ver. 20). All tests were conducted at a 95% confidence interval, and values of p < 0.05 and p < 0.001 were considered statistically significant.
