# Supplementary material for: Tracing geochemical sources and health risk assessment of uranium in groundwater of arid zone of India
Source: Sci Rep. 2022 Jun 1;12:2286. doi: 10.1038/s41598-022-05770-2 (PMC9160070; doi:10.1038/s41598-022-05770-2)
Supplement: Supplementary file 2 — Supplementary Information 2. [file 41598_2022_5770_MOESM2_ESM.docx]

**Supplementary text (S2)**

**Tracing Geochemical Sources and Health Risk Assessment of Uranium in Groundwater of Arid Zone of India.**

Pragya Pandit^1, 2^, Atul Saini^3^, Sabarathinam Chidhambaram^4^, Vinod Kumar^5^, Banjarani Panda^6^, Ramanathan A.L.^7^, Netrananda Sahu^2^, A. K. Singh^8^

^1^Atomic Minerals Directorate for Exploration and Research, New Delhi-110066, India.

^2^Phd, USICT, GGSIPU, Dwarka, Delhi, 110078.

^3^Department of Geography, Delhi School of Economics, University of Delhi, Delhi -110007, India

^4^Water Research Center, Kuwait Institute for Scientific Research, Kuwait

^5^ Department of Botany, Government Degree College, Ramban-182144, Jammu, India

^6^ Water Sciences Lab, University of Nebraska-Lincoln, Lincoln, Nebraska, USA.

^7^School of Environmental Sciences, Jawahar Lal Nehru University, New Delhi-110067

^8^Maharaja Surajmal Institute of Technology, USICT, GGSIPU, New Delhi 110058, India

**Table S1 Health risk associated with uranium in drinking water**

| Parameter | Description |
| --- | --- |
| Radiological risk assessment  $CancerRisk=A_{c}xR$ | The cancer mortality risk coefficients for the three isotopes of uranium namely ^234^U, ^235^U and ^238^U were taken as 6.1 x 10^-11^, 6.2 x 10^-11^ and7.5 x 10^-11^$B{qL}^{-1}$ while cancer morbidity risk coefficients were 9.5 x 10^-11^, 9.8 x 10^-11^ and 7.5 x 10^-10^$B{qL}^{-1}$ respectively. There are three naturally occurring isotopes of uranium with $T_{1/2}$ 2.4 x 10^5^, 7.0 x 10^8^ and 4.47 x 10^9^ years for ^234^U, ^235^U and ^238^U. United States Environmental Protection Agency (USEPA, 2000) proposed the method to assess radiological risk due to the ingestion of uranium in water. Here A_C_ is the activity concentration of uranium in) and R is the Risk factor ($B{qL}^{-1}$) which is a product of risk coefficient and per capita activity intake. |
| Chemical toxicity risk estimation  $\text{LADD}=\frac{\text{EPC}\times\text{IR}\times\text{IF}\times D}{\text{AT}\times W}$  $\text{HQ}=\frac{\text{LADD}}{\text{RFD}}$ | The chemical/non-carcinogenic risk for uranium was quantified in terms of lifetime average daily dose (LADD) and hazard quotient (HQ). Where EPC is the exposure point concentration of uranium in water (µg L^-1^), IR = ingestion rate (1.4 $LD^{-1}$) ), IF = ingestion frequency (365 days year^-1^), D = Duration (69.89 years), AT = average time (days) and W = ideal body weight (68.831 kg) (ICRP 2014).  RFD is the reference dose limit taken as 0.6 l $mg{kg}^{-1}{day}^{-1}$ (WHO 2004). |
| Annual ingestion dose  $D_{2}=A_{c}xWIxDCF$ | Ac is the activity concentration of uranium in the water sample ($B{qL}^{-1}$) calculated using mass to an activity conversion factor of 0.025, WI is annual water intake (L year-1) and DCF is the dose conversion factor ($Sv{Bq}^{-1}$). The daily water intake through drinking water for each age-group in accordance with the Institute of Medicine of the National Academies, Washington (2005) was computed in the present study. The dose conversion factors (DCFs) prescribed for infants, children of 1, 5, 10 and 15 years, and adults (> 18 years) are 3.4 x 10^-7^, 1.2 x 10^-7^, 8 x 10^-8^, 6.8 x 10^-8^, 6.7 x 10^-8^ and 1.3 x 10^-8^ ($Sv{Bq}^{-1}$) respectively (ICRP 2012; Li et al. 2009). |
| Dose assessment to different organs using Hair compartment model of uranium  $\frac{{dq}_{i}}{dt}=\sum_{i} \lambda_{ji}q_{i}-\left( \lambda_{i}+\lambda_{r} \right).q_{i},i,j,\ldots,n$ | The first-order differential equation describes the activity movement of uranium between compartments. Where dq_i_ is the change in the amount of uranium (in units of mass) in a particular compartment, dt is the small-time interval, λ_ji_ is transfer rate between two specific compartments (from compartment j to i), q_i_ is transfer rate from i^th^ compartment, λ_r_ is the rate of radioactive decay and n is the number of compartments. After ingestion via drinking water, all absorption of uranium occurs in the small intestine (SI) with alimentary tract transfer factor (f_1_) as 0.6%. From SI, the uranium enters plasma and then distributed and reabsorbed from soft tissue, liver, skeleton and kidneys. The hair compartment model, likeICRP’s Biokinetic Model has a recycling nature |

**Table S2 Summary of exposure assumptions used to calculate health risk assessment**

| Exposure Route/Exposure Factor | Symbol | Value | | | | Units | Reference/Source |
| --- | --- | --- | --- | --- | --- | --- | --- |
| Water ingestion rate-adult | IRW_a_ | 1.4 | | | | L/day | (Rani et al .2013; Harries and Harper 2004) |
| Water ingestion rate-child | IRW_c_ | 0.64 | | | | L/day | (Harries and Harper 2004) |
| Contaminant concentration in groundwater | C_w_ | specific | | | | µg/l |  |
| Exposure frequency | EF | 365 | | | | Days/year |  |
| Exposure duration-adult | ED_a_ | 30 | | | | Year | (Harries and Harper 2004) |
| Exposure duration-child | ED_c_ | 6 | | | | Year | (Harries and Harper 2004) |
| Body weight-adult | BW_a_ | 70 | | | | Kg | (Rani et al. 2013;Harries and Harper 2004) |
| Body weight-child | BW_c_ | 15 | | | | Kg | (Harries and Harper 2004) |
| Average time-adult | AT_a_ | 10950 | | | | Days | AT= EF x ED |
| Average time-child | AT_c_ | 2190 | | | | Days | AT= EF x ED |
| Absorbed dose per event | DA_event_ | Calculated value | | | | Mg/cm^2^-event |  |
| Exposure time-adult | ET_a_ | 0.58 | | | | Hours/day | (USEPA 2004) |
| Exposure time-child | ET_c_ | 1 | | | | Hours/day | (USEPA 2004) |
| Event frequency-adult | EV_a_ | 1 | | | | Event/day | (USEPA 2004) |
| Event frequency-child | EV_a_ | 1 | | | | Event/day | (USEPA 2004) |
| Skin surface area-adult | SA_a_ | 18000 | | | | Cm^2^ | (USEPA 2004) |
| Skin surface area-child | SA_c_ | 6600 | | | | Cm^2^ | (USEPA 2004) |
| Conversion factor | CF | 0.001 | | | | L/cm^3^ | 1L=1000 cm^3^ |
| Dermal permeability coefficient | K_p_ | Contaminant of potential concern (Pb, Cu, V and Li= 0.001, Zn= 0.0001, Ni= 0.0002, Co= 0.0004 | | | | Cm/hour | (USDOE 2011 ) |
| Reference dose-ingestion/dermal | RfD_ing/der_ | Met-al | RF_der_ | | RF_ing_ | mg/kg/day | (USDOE 2011), Equation 6 |
|  |  | Pb  Cu  Zn  Ni  Co  V  Li | 1.4  40  300  20  0.3  7  0.42 | 1.4  8  60  0.8  0.06  7  0.42 | |  |  |
| Cancer slope factor-ingestion | CSF_0_ | Ni= 0.84, Pb= 8.5 | | | | (mg/kg/day)^-1^ | (USDOE 2011) |
| Chronic daily intake-non-carcinogenic ingestion | CDI_ing-nc_ | Calculated value | | | | mg/kg/day | Equation 4 |
| Chronic daily intake-non-carcinogenic dermal | CDI_derm-nc_ | Calculated value | | | | mg/kg/day | Equation 5 |
| Chronic daily intake-carcinogenic-ingestion | CDI_ing_-_cr_ | Calculated value | | | | mg/kg/day | Equation 7 |
| Chronic daily intake-carcinogenic-dermal | CDI_derm_-_cr_ | Calculated value | | | | mg/kg/day | Equation 7 |

| **Table S3** Principal component analysis of physic-chemical and heavy metals in groundwater from Rajasthan | | | | |
| --- | --- | --- | --- | --- |
| **Rotated component matrix** | | | | |
| **Variables** | PC1 | PC2 | PC3 | PC4 |
| **pH** | -0.05 | -0.18 | **-0.79** | -0.15 |
| **Cond.** | **0.97** | 0.06 | -0.01 | -0.06 |
| **TDS** | **0.96** | 0.08 | -0.01 | -0.07 |
| **Na** | **0.93** | 0.12 | -0.07 | 0.06 |
| **K** | 0.21 | -0.14 | **0.45** | 0.06 |
| **Ca** | **0.51** | -0.40 | **0.47** | 0.20 |
| **Mg** | **0.65** | -0.39 | 0.28 | -0.05 |
| **Cl** | **0.94** | -0.16 | 0.07 | 0.08 |
| **HCO_3_** | -0.02 | **0.86** | 0.07 | -0.11 |
| **SO_4_** | **0.71** | -0.17 | 0.26 | 0.15 |
| **PO_4_** | 0.04 | 0.04 | 0.04 | -0.06 |
| **F** | 0.02 | 0.43 | 0.10 | **0.56** |
| **V** | -0.08 | **0.60** | 0.03 | 0.09 |
| **Li** | -0.12 | 0.13 | -0.20 | **-0.62** |
| **Co** | 0.02 | 0.10 | -0.24 | 0.04 |
| **Ni** | -0.05 | 0.03 | 0.10 | **0.65** |
| **Cu** | -0.05 | -0.11 | 0.10 | -0.03 |
| **Zn** | -0.11 | 0.07 | 0.17 | -0.14 |
| **Pb** | -0.04 | -0.01 | **0.44** | **-0.48** |
| **U** | **0.51** | **0.50** | -0.18 | -0.05 |
| **% Var** | 25.66 | 10.60 | 7.95 | 7.51 |

**Table S4** Doses to various organs and tissues of a human adult from ingested uranium via drinking water pathway

| **Organs** | **Mean** | **Median** | **Min** | **Max** | **I Quartile** | **III Quartile** | **IQR** | **Range** | **Std. Dev** |
| --- | --- | --- | --- | --- | --- | --- | --- | --- | --- |
| **Adrenals (µSv)** | 1.801 | 1.432 | 0.038 | 19.446 | 0.754 | 2.487 | 1.734 | 19.408 | 1.644 |
| **Bladder Wall (µSv)** | 1.801 | 1.432 | 0.038 | 19.446 | 0.754 | 2.487 | 1.734 | 19.408 | 1.733 |
| **Bone Surfaces (µSv)** | 51.882 | 41.263 | 1.086 | 560.312 | 21.718 | 71.668 | 49.950 | 559.225 | 47.375 |
| **Brain** | 1.801 | 1.432 | 0.038 | 19.446 | 0.754 | 2.487 | 1.734 | 19.408 | 1.644 |
| **Breasts (µSv)** | 1.801 | 1.432 | 0.038 | 19.446 | 1.754 | 1.131 | 1.734 | 19.408 | 1.644 |
| **St Wall (µSv)** | 2.106 | 1.675 | 0.044 | 22.742 | 0.881 | 1.322 | 2.027 | 22.698 | 1.923 |
| **SI wall (µSv)** | 2.533 | 2.015 | 0.053 | 27.356 | 1.060 | 3.500 | 2.439 | 27.303 | 2.313 |
| **ULI wall (µSv)** | 6.409 | 5.097 | 0.134 | 69.215 | 2.683 | 21.500 | 6.170 | 69.080 | 6.170 |
| **LLI wall (µSv)** | 15.565 | 12.379 | 0.326 | 168.093 | 6.517 | 25.716 | 14.985 | 167.768 | 14.213 |
| **Kidneys (µSv)** | 18.617 | 14.806 | 0.390 | 201.053 | 7.793 | 9.696 | 17.923 | 200.663 | 16.994 |
| **Liver (µSv)** | 7.019 | 5.583 | 0.147 | 75.807 | 2.938 | 2.487 | 6.758 | 19.408 | 1.644 |
| **Lungs (µSv)** | 1.801 | 1.432 | 0.038 | 19.446 | 0.754 | 2.487 | 1.734 | 19.408 | 1.644 |
| **Muscle (µSv)** | 1.801 | 1.432 | 0.038 | 19.446 | 0.754 | 2.487 | 1.734 | 19.408 | 1.644 |
| **Ovaries (µSv)** | 1.801 | 1.432 | 0.038 | 19.446 | 0.754 | 2.487 | 1.734 | 19.408 | 1.644 |
| **Pancreas (µSv)** | 1.801 | 1.432 | 0.038 | 19.446 | 0.754 | 2.487 | 1.734 | 19.408 | 5.102 |
| **Red Marrow (µSv)** | 5.493 | 4.369 | 0.115 | 59.327 | 2.300 | 7.498 | 5.289 | 59.212 | 1.644 |
| **Skin (µSv)** | 1.801 | 1.432 | 0.038 | 19.446 | 0.754 | 5.226 | 1.734 | 19.408 | 1.644 |
| **Spleen (µSv)** | 1.801 | 1.432 | 0.038 | 19.446 | 0.754 | 5.846 | 1.734 | 19.408 | 1.644 |
| **Testes(µSv)** | 1.770 | 1.408 | 0.037 | 19.117 | 0.741 | 7.033 | 1.704 | 19.079 | 1.616 |
| **Thymus (µSv)** | 1.801 | 1.432 | 0.038 | 19.446 | 0.754 | 17.794 | 1.734 | 19.408 | 1.644 |
| **Thyroid (µSv)** | 1.801 | 1.432 | 0.038 | 19.446 | 0.754 | 2.487 | 1.734 | 19.408 | 1.644 |
| **Uterus (µSv)** | 1.801 | 1.432 | 0.038 | 19.446 | 0.754 | 2.487 | 1.734 | 19.408 | 1.644 |
| **Remainder Dose (µSv)** | 12.326 | 4.567 | 0.083 | 70.860 | 1.163 | 19.488 | 18.326 | 70.785 | 14.376 |
| **Effective Dose (µSv)** | 4.710 | 3.863 | 0.114 | 41.791 | 1.944 | 6.479 | 4.534 | 41.677 | 4.091 |

.


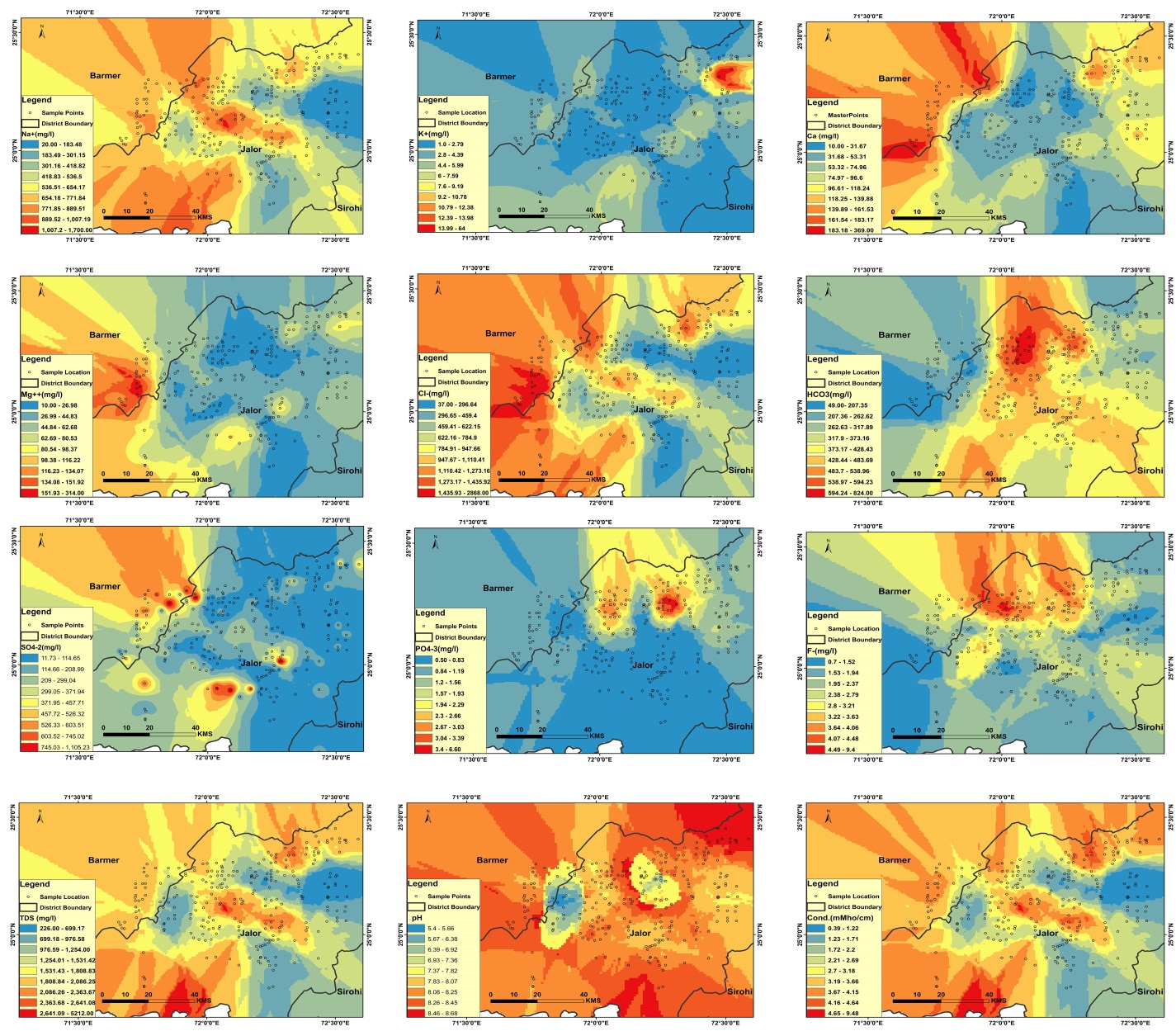


**Fig. S1** Spatial Distribution of Physico-chemical parameters in Rajasthan and Gujarat.

The spatial plot was drawn using Arc GIS 10.1.


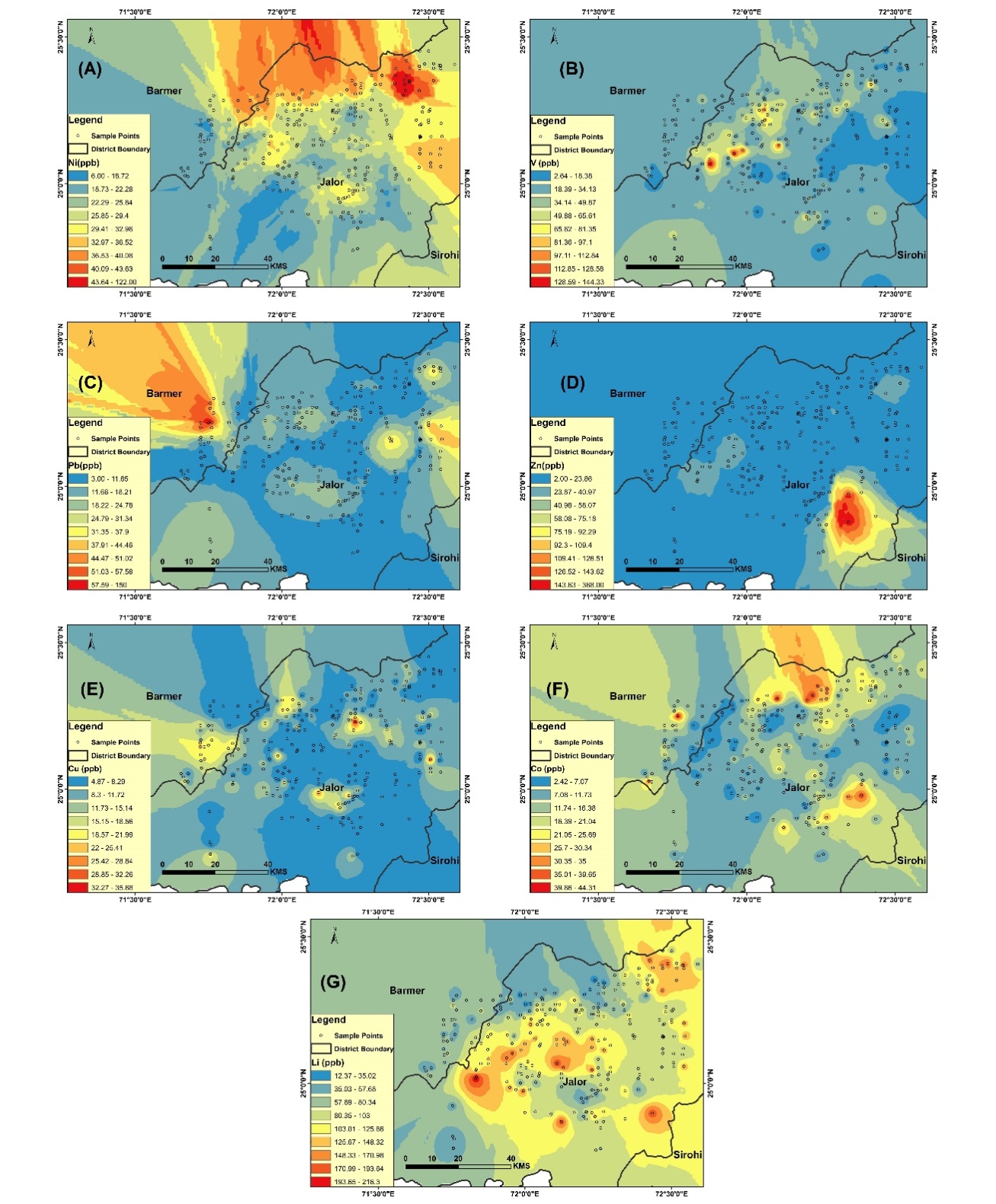


**Fig. S2** Spatial distribution of heavy metals in districts of western Rajasthan and Gujarat.

The spatial plot was drawn using Arc GIS 10.1.


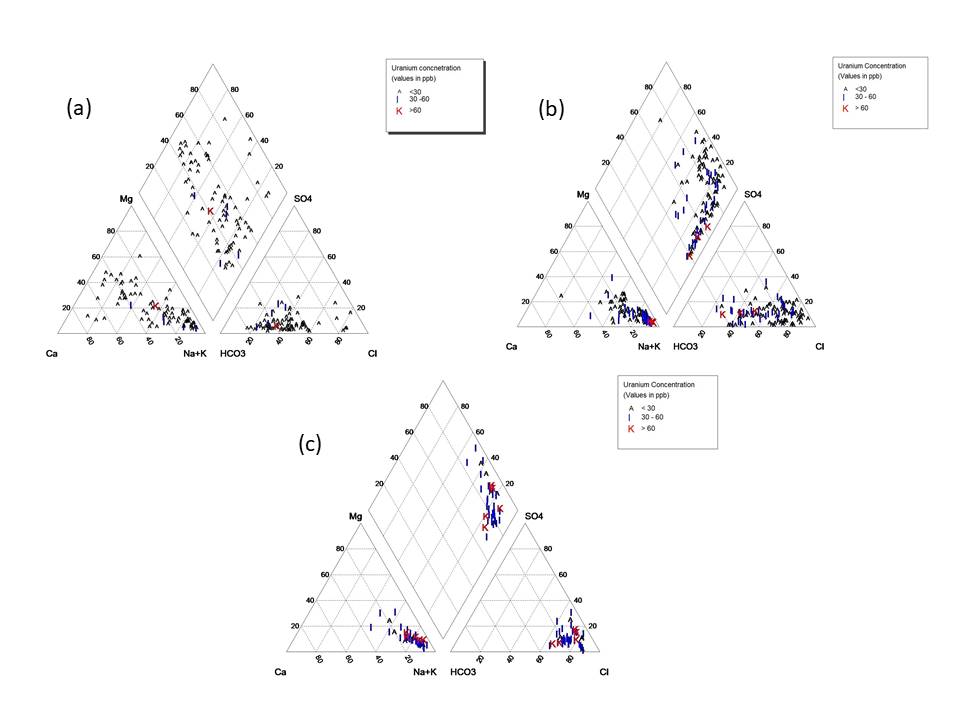


**Fig S3 Piper plots indicating the hydrogeochemical facies of U<30 ppb, 30<U<60 ppb and U >60 ppb in (a) TDS<1000 mgL^-1^ (b) 1000 <TDS<2000 mgL^-1^ (c) TDS>2000 mgL^-1^**

**The** **spatial plots were drawn using Aqua Chem ver 9.0 software.**
