# Supplementary material for: Tracing geochemical sources and health risk assessment of uranium in groundwater of arid zone of India
Source: Sci Rep. 2022 Jun 1;12:2286. doi: 10.1038/s41598-022-05770-2 (PMC9160070; doi:10.1038/s41598-022-05770-2)
Supplement: Supplementary file 3 — Supplementary Information 3. [file 41598_2022_5770_MOESM3_ESM.docx]

**Supplementary text (S3)**

**Tracing Geochemical Sources and Health Risk Assessment of Uranium in Groundwater of Arid Zone of India.**

Pragya Pandit^1, 2^, Atul Saini^3^, Sabarathinam Chidhambaram^4^, Vinod Kumar^5^, Banjarani Panda^6^, Ramanathan A.L.^7^, Netrananda Sahu^2^, A. K. Singh^8^, Rohit Mehra9

^1^Atomic Minerals Directorate for Exploration and Research, New Delhi-110066, India.

^2^Phd, USICT, GGSIPU, Dwarka, Delhi, 110078.

^3^Department of Geography, Delhi School of Economics, University of Delhi, Delhi -110007, India

^4^Water Research Center, Kuwait Institute for Scientific Research, Kuwait

^5^ Department of Botany, Government Degree College, Ramban-182144, Jammu, India

^6^ Water Sciences Lab, University of Nebraska-Lincoln, Lincoln, Nebraska, USA.

^7^School of Environmental Sciences, Jawahar Lal Nehru University, New Delhi-110067

^8^Maharaja Surajmal Institute of Technology, USICT, GGSIPU, New Delhi 110058, India.

9B. R. Ambedkar National Institute of Technology, Jalandhar, 144011, India.

**Results and Discussion**

**Spatiotemporal variation of major ions**

The spatial distributions of the major cations and anions are depicted in **Fig. S1**. A comparison against the permissible limits of BIS (2012) and WHO (2017) indicated that almost 71% of the samples had pH <8.5. Accordingly, progressive evaporation leads to enhancement in salinity and alkalinity. TDS values indicated 224–5212 mgL^-1^  from 56% of the samples, markedly below the permissible limits by BIS. By and large, TDS levels increased towards west to southwest direction near the margins of Ranns, i.e., Gandhav (767 mg L^-1^) and Arwa (1884 mgL^-1^). According to Yadav and Sarin (2009), this is due to the release from weathering of calcareous rocks and atmospheric aerosol transport of salt during summer from Rann of Kutch as a source of salt to the lake basin. A natural resources survey by the Centre for Arid Zone Research Institute (CAZRI) inferred that extensive calcrete occurrences are sub-soil features or as an outcrop under thin aeolian sand covers (Dhir et al., 2004). On the whole, it could be said that there has been an increase of salinity towards west and southwest, which in turn, is directly related to aridity conditions and mixing of waters with Rann of Kutch. Similar to TDS, conductivity has a parallel distribution pattern. Regarding physicochemical parameters, the relative abundance of major cations observed was Na^+^ > Ca^2+^ > Mg^2+^ > K^+^. Na^+^ was seen to be the most dominant cation, with concentration ranging from 20 to 1700 mg L^-1^ (mean value 585.36 mg L^-1^) > K^+^ (3.75 mgL^-1^), Ca^2+^ (88.6 mgL^-1^**)** and Mg^2+^ (49.6 mgL^-1^**)**. Only 18% of the samples had Na^+^ values below the permissible limits of drinking water, prescribed by BIS (2012). The presence of Na^+^ could be partly due to geogenic (water-rock) interaction. Almost 94% of the K^+^ samples and 93% of the Ca^2+^ had values below BIS permissible limits. Potassium levels were detected to be of very low order in all channels (1–64 mgL^-1^), because the degree of weathering may not be matured enough for disintegration and decomposition of feldspars. Simultaneously, 90% of the Mg^2+^ and 69% of the Cl^-^ samples had values below the permissible limits of BIS (2012). The spatial distribution of Na^+^, Ca^2+^, and Cl^-^ (1435.93–2868 mgL^-1^) showed a similar pattern, having high values in the north, northwest, west, and southwest directions and low values in the middle. This relationship was detected to be also obvious by the Pearson correlation coefficient.

The anions followed the trend Cl^-^ > HCO_3_^-^ > SO_4_^2-^ > CO_3_^2-^ > PO_4_^3-^ > F^-^. The data for F^-^ followed normal statistics from 0.7–9.4 mgL^-1^, and the mean value observed was 2.67 mgL^-1^**.**  Comparative analysis of F^-^ with values from other locations reported that 74% of groundwater samples had F- content above the WHO limit of 1.5 mgL^-1^. Groundwater with F^-^ content exceeding the WHO/BIS permissible limit was seen mostly occurring in the north direction (4.09–9.4 mgL^-1^), indicative of felsic igneous provenance. Incidentally, this value is higher than those reported by earlier studies in Pali, Jalore, and Barmer district of Rajasthan (Singh et al., 2011). High pH as well as alkalinity and low levels of Ca, Mg, and total hardness would imply favourable chemical conditions for the fluoride dissolution process. Correspondingly, high Cl^-^ concentration revealed that origin of Cl^-^ is both geogenic and anthropogenic (Chidambaram et al., 2007). The 89% samples of the SO_4_^2-^ showed to have values below BIS prescribed limits. It is worth noting that SO_4_^2-^ contents are mainly due to the occurrence of barium sulfate (BaSO_4_) in the form of veins associated with volcanic agglomerate in MIS.

**Spatial distribution of metals**

In the study area, Pb concentration varied from 3–150 µgL^-1^ with an average value of 15.9 µgL^-1^, which is close to the acceptable limit. The acceptable limit for Pb is 10 µgL^-1^ (WHO, 2017), and the world’s average concentration of Pb is 0.08 µgL^-1^. On the other hand, the acceptable limit of copper in drinking water is 15,000 µgL^-1^ (WHO, 2017). Results showed that 100% of the samples was within the margin of the WHO limit. The permissible limit of Zn is 3000 µgL^-1^ (WHO, 2017). It was found that 100% of the samples showed below the permissible limits of both BIS (2012) and WHO (2017). Water pipes having Zn metal carrying water leads to enhanced Zn in the groundwater. A mild positive correlation of Zn was observed with Li.

With reference to cobalt (Co), the average value of dissolved Co was 12.8 µg L^-1^. The world’s average value for Co is 0.9 µgL^-1^. All of the samples have values above the world’s average. Incidentally, Co is an independent mineral with no correlation with major cations and other elements. The likely source of Co is due to the dissolution of the bedrock minerals. Conversely, when referring to Cu, its likely source is identified to be mostly anthropogenic, since no major Cu mineral was identified in the saturation indices. Ni (0.658) showed a positive correlation with F^-^ (0.378); hence, the source of Ni is geogenic, due to the dissolution of minerals, retgersite (NiSO_4_.6H_2_O) and morenosite (NiSO_4_.7H_2_O). Average Li concentration was <100 µg L^-1^ and in general, is associated with brackish to brine waters (Bhandary et al., 2018). V concentration, on the other hand, ranged from 2 to 156 µg L^-1^ with a mean value of 29.76 µg L^-1^. The anomalous V concentration in groundwater could be related to carnotite mineralization weathering. Spatial distribution is essential for evaluating spatiotemporal evaluation of water quality and hazardous parameters (Duan et al., 2013). The spatial distributions of base metals Zn, Cu, Pb, Li, V, Co are shown in **Fig. S2.** The spatial distribution indicated the concentration of Ni in the northern direction (43.64–122 µg L^-1^); V was seen to be normally uniformly distributed in all the directions with high values in the center (128.59–144.33 µg L^-1^). Pb showed a high concentration in the northwest direction (57–150 µg L^-1^). This enhancement in northwest direction is due to the fact that northwest of Bhinmal, Jalore District along Sagi-Sukri channels has an abundance of minerals, such as limestone, gypsum, jasper, garnet, marbles, lead, zinc, phophatic rock, gypsum, and clay. Alternatively, Zn had a high concentration in the southeast direction (413.63–368 µg L^-1^). Cu was observed to be uniformly distributed spatially (4–8 µg L^-1^). Co was, on the one hand, detected to have enhancement in the northern direction. Contrariwise, Li was noted to be clearly concentrated in high value in the centre, northeast, and southwest directions (103–125.6 µg L^-1^) as illustrated in **Fig. S2.** Hydrogeochemical analysis of the samples was carried out to establish the source of major cations and anions in the solution.

**Geochemical facies of groundwater:**

Na-Cl-SO_4_, Na-Cl-HCO_3_, Na-Cl, Na-Mg-HCO_3_, Ca-Mg-Cl-HCO_3_, Na-Ca-Cl-SO_4_, Na-HCO_3_, Na-Mg-Cl, Mg-Na-Ca-Cl, Na-Mg-Cl-SO_4_, Na-Ca-Cl, Na-Cl Mg-HCO_3_-Cl, Na-Ca-Cl-HCO_3_, Na-Cl-HCO_3_, Na-HCO_3_ were the different hydrochemical facies observed in the study area. The analytical results obtained from the TDS1 group revealed that alkali group elements (Na^+^ + K^+^) were seen to have exceeded the alkaline group elements (Ca^2+^ + Mg^2+^) in 89% of the samples. Equally, 60% of the samples exhibited the dominance of the weak acid (Cl^-^ + SO_4_^2-^) over the strong acids (HCO_3_^-^). Hence, the predominant hydrochemical facies obtained in this TDS1 group were Na-HCO_3_^-^--Cl^-^ and Na-Mg-HCO_3_, where 37% of the samples belong to this group. These samples are typical of coastal aquifer system unaffected by salinization. A correlation analysis study indicated that in the first group, TDS was analysed to have strong correlation with conductivity (r = 0.954, p<0.05) and Na (r = 0.819, p<0.05), although having poor correlation with U (r = 0.430, p<0.05). Only 26% of the group 2 samples, TDS 2 (1000<TDS<2000 mgL^-1^) were detected to belong to Na-HCO_3_-Cl. These samples have enhanced salinity due to surface evaporation, resulting from the capillary rise method. TDS 2 was perceived having mild positive correlation with Na^+^ (r= 0.665, p<0.05), Ca^2+^(r=0.271, p<0.05), Mg^2+^ (r= 0.335, p<0.05) and SO_4_^2-^ (r= 0.446, p<0.05). In TDS 3 group, the major hydrochemical facies observed were Na–Cl and Na–Mg–Ca–Cl. At this juncture, about 61% of the samples were seen to belong to the hydrochemical facies, Na-Cl type. Over again, it may be said that a significant correlation was noted to exist between Na^+^ and Cl^-^ (r= 0.620, p<0.05); and TDS, having strong positive correlation with conductivity (r =0.979, p <0.05); mild positive correlation with U (r= 0.473, p<0.05); and Cl (r =0.761, p<0.05).

**Silicate Weathering and Ion Exchange Process**

Weathering plots are used to estimate the relative contribution of cations and anions to ionic process. Na^+^/C1^-^ ratios are an important indicator of sources of salinity during groundwater flow and signify the role of silicate weathering, cation exchange, or halite dissolution (Tiwari et al., 2019). The plot of Na^+^/Cl^-^ showed that several samples fell near the equiline of 1:1, denoting halite dissolution or silicate weathering **Fig. 2(a).** The Na^+^/C1^-^ rations were seen to lie between 0.20 and 4.7 with an average of 1.41. As per our study, 68% of Na^+^/Cl^-^ molar ratio were greater than 1, The Na^+^/C1^-^ ratios of >1 could be typically interpreted as Na^+^ released from a silicate weathering and can be attributed to the interaction of groundwater with feldspathic schist/soda granite. Cl-/Sum of anions using rock source deduction of Aquachem software identified evaporites and halite dissolution to be the main source for Cl^-^ ions.

Ca^2+^/Mg^2+^ ratio has been used to determine the sources of calcium and magnesium ions into the groundwater environment. The majority of samples (56%) had a Ca^2+^/Mg^2+^ ratio of less than 1 and 44% had a ratio greater than 1 (**Fig. 2(b)**)**.**  The Ca^2+^ and Mg^2+^ content in the groundwater could be attributed to base exchange reactions, where chlorite schist and basic rocks are the dominant aquifer lithology, signifying that most of the samples are remote from the 1:1 line, implying that carbonate dissolution and gypsum dissolution are not the main causes of Ca^2+^ and Mg^2+^, but rather the main source could be ion. Since the majority of the samples were observed to fall below the ratio line, this could signal precipitation of Ca^2+^ as CaCO_3_, resulting in a decline of Ca^2+^ values or ion exchange process. The chemical data of groundwater samples were plotted for Na^+^ +K^+^ )and Ca^2+^ + Mg^2+^ vs Tz (total cations) and depicted in **Fig 2(c) and Fig 2(d).** The graphs show that most of the samples fall close to or below the 1:1 theoretical line, inferring the supply of cations via ion exchange **(Thivya et al., 2013).** The relatively low mean ratio of Ca^2+^+ Mg^2+^/Total Cation, Tz (0.27), and high ratio of Na^+^+K^+^/Tz (0.72) would suggest that carbonate dissolution is less intense when compared to silicate weathering and evaporite dissolution. It is worth noting that the plot of (Ca^2+^ +Mg^2+^) versus (HCO_3_^-^ + SO_4_^2-^) has been used to establish the ion exchange processes (**Fig 2(e)**). If ion exchange were dominant, it would tend to shift to the right due to an excess of HCO_3_^-^ + SO_4_^2-^. Contrariwise, if reverse ion exchange were the process used, they would shift to the left, owing to an excess of Ca^2+^ + Mg^2+^ over HCO_3_^-^ + SO_4_^2-^. The points falling near 1:1 equiline suggested silicate weathering sources. The study therefore would infer that 48% of Ca^2+^ +Mg^2+^ has a ratio less than 1, and 52% ratio greater than 1 **Fig. 2(f),** demonstrating the dominance of ion exchange process and that, most of the higher U samples fall in this region. The Na^+^+ K^+^  plotted against SO_4_^2^ + Cl^-^ showed the majority of the samples lying close to the 1:1 equiline, suggesting mineral dissolution as the major process controlling the ion chemistry as illustrated in **Fig 2(g).**

The difference between the Ca^2+^ + Mg^2+^ and HCO_3_^-^ + SO_4_^2-^ was plotted against the difference between Na^+^ and Cl^-^. The plot was seen to depict four basic fields, depending upon the dominance of Na, Cl, Ca^2+^ +Mg^2+^ and HCO_3_^-^+ SO_4_^2-^. **Fig 2(g)** presents that nearly 95% of the samples reveal higher U plot in the region with a relative greater concentration of Na and HCO_3_^-^ + SO_4_^2-^. The same **Fig 2(g)** shows a negative line with a slope of unity with slight dispersion, indicative of ion exchange to be the dominant process controlling water-rock interaction. Markedly**,** It was observed that most of the samples with U>30 ppb had a relatively higher Na^+^ concentration than Cl^-^.

Accordingly, weathering plots revealed ion exchange, silicate weathering, and evaporate dissolution to be the main hydrogeochemical process contributing to ionic load.

**Carnotite saturation index**

On the other hand, carnotite solubility index (CSI) is another measure of solubility of the state of equilibrium between groundwaters and carnotite mineralization which may be presented in channels. A number of mechanisms, including sorption, colloidal precipitation, change in vanadium redox state and in CO_2_ partial pressure (pCO_2_), and pH have been proposed to explain the precipitation of carnotite. Carnotite precipitation takes place in the range of 700 to 800 ppm potassium values. The corresponding formula of CSI is as follows:

$I=\frac{\log\left( U \right)\left( V \right)\left( K \right)}{1.13X{10}^{4}}$ (10)

When the CSI is zero: Carnotite in the equilibrium state.

CSI is –ve: There is a tendency to dissolve (undersaturated).

CSI is +ve: Tendency to be saturated to supersaturated/ precipitate.

CSI varying between -3.89 and -8.30 is quite high, which infers that carnotite is undersaturated in the waters. A further implication is the dissolution of U in water. However, earlier, low solubility about 3 × 10^−7^ M L^−1^ at 25°C and pH 7.0–8.0 has been reported for carnotite **(Langmuir ,1978).**
